# Engineered live bacteria for liver diseases and gut-liver axis disorders: from genetic modification to advanced delivery systems

**DOI:** 10.1186/s12951-026-04483-2

**Published:** 2026-04-29

**Authors:** Yanan Zhang, Yawen Zhu, Yun Yang, Dayu Chen, Jinglin Wang

**Affiliations:** 1 Division of Hepatobiliary and Transplantation Surgery, Department of General Surgery, Nanjing Drum Tower Hospital, Clinical College of Nanjing University of Chinese Medicine, Nanjing, 210008 China; 2 Department of Pharmacy, Nanjing Drum Tower Hospital, Affiliated Hospital of Medical School, Nanjing University, Nanjing, 210008 China; 3 Department of Stomatology, Nanjing Drum Tower Hospital, Affiliated Hospital of Medical School, Nanjing University, Nanjing, 210008 China

**Keywords:** Liver diseases, Engineered live bacteria, Gut-liver axis, Drug delivery, Therapeutics

## Abstract

Liver diseases, including hepatocellular carcinoma (HCC), non-alcoholic fatty liver disease (NAFLD), and alcoholic liver disease (ALD), impose a significant global health burden, with over 2 million deaths annually and substantial economic losses. Current treatments, primarily pharmacological, face challenges such as insufficient efficacy, poor absorption, and drug resistance. The liver-gut axis, a critical pathway linking the liver and intestines, offers a therapeutic target for these diseases. Engineered live bacteria, modified through genetic engineering and synthetic biology, have emerged as a promising alternative. These bacteria can be designed to deliver therapeutic agents directly to the liver or gut, enhancing efficacy and reducing systemic side effects. This review explores the application of engineered live bacteria in treating liver diseases, focusing on strains such as *Bifidobacterium*, *Escherichia coli Nissle 1917* (EcN), *Bacillus subtilis* (*B. subtilis*), *Saccharomyces boulardii* (*S. boulardii*), and *Lactobacillus reuteri* (*L. reuteri*). It also pays attention to the internal genetic modification and external covalent connection of the original live bacteria. We discuss the design of dosage forms, including capsule formulations, microencapsulation, and nanopreparations, and administration methods like oral and in situ injection. Additionally, we address the challenges and future prospects of using engineered live bacteria to target liver diseases and related conditions, aiming to advance their clinical application and reduce the global burden of liver diseases.

**Graphical Abstract:**

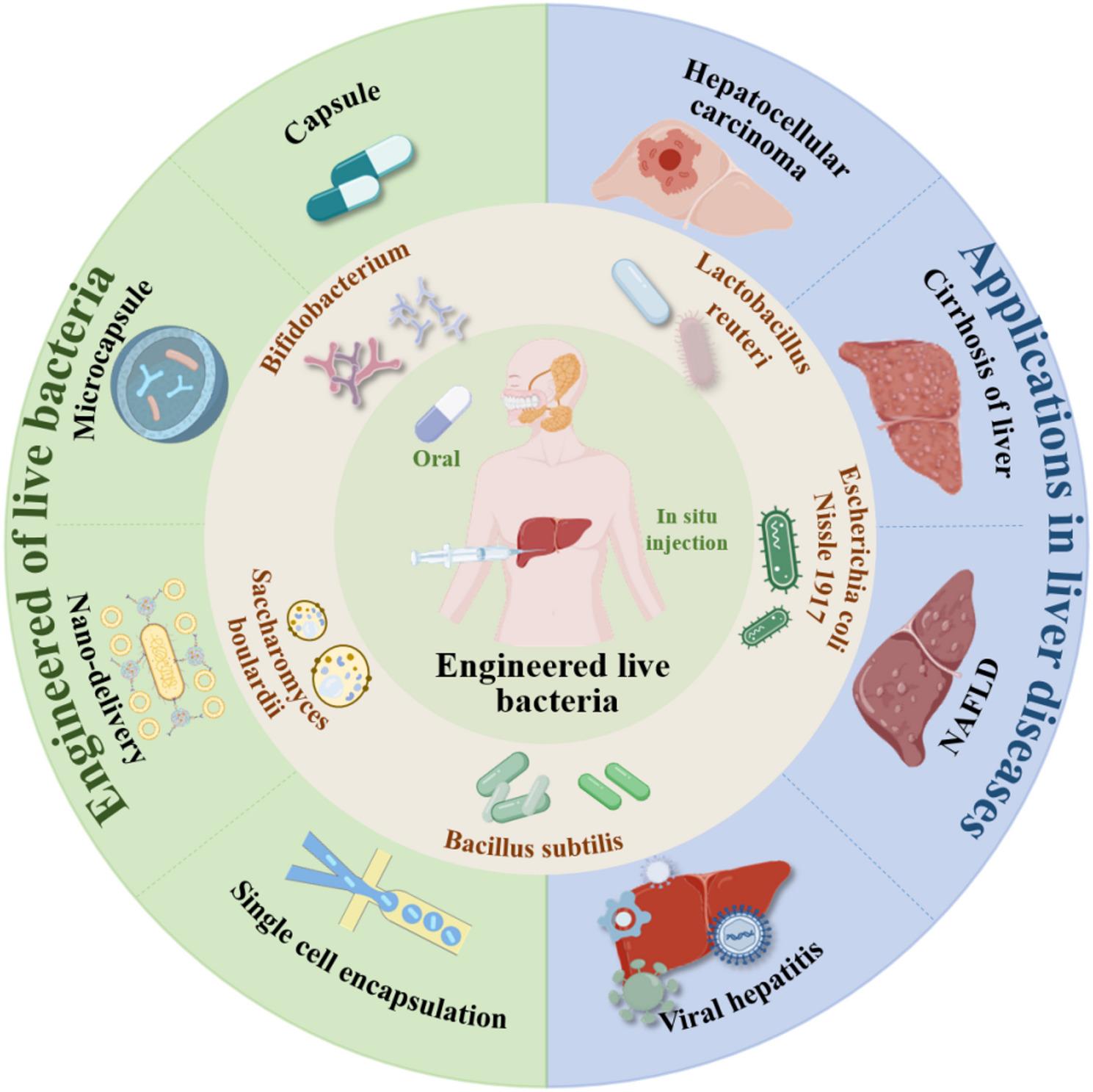

## Introduction

Liver diseases represent a significant global health challenge, affecting millions of individuals and contributing to over 2 million deaths annually [1]. These diseases encompass a wide spectrum of conditions, including HCC, NAFLD, ALD, viral hepatitis, and cirrhosis. The economic burden associated with liver diseases is substantial, with healthcare costs exceeding $120 billion in the United States alone [2]. Current treatment strategies primarily rely on pharmacological interventions, such as antiviral drugs for viral hepatitis and antitumor drugs for HCC. However, these treatments often suffer from limitations such as insufficient efficacy, poor absorption, inability to maintain therapeutic blood concentrations, and the development of drug resistance and side effects [3]. As a result, developing innovative therapeutic strategies that demonstrate robust efficacy alongside reduced side effects is thus a critical priority.

The gut-liver axis, a system of reciprocal interaction between the gut and the liver that drives both disease initiation and advancement. This axis facilitates the transfer of intestinal metabolites, immune cells, and microbial products to the liver, influencing liver function and health [4]. An imbalance in the gut microbial community is recognized as driver in the pathogenesis of a spectrum of hepatic conditions, such as NAFLD, ALD, and HCC. Therefore, a novel frontier in hepatology involves the therapeutic targeting of both the gut microbiome and the gut-liver axis, an approach that is increasingly recognized for its potential [5, 6]. Engineered live bacterial systems constructed through genetic modification and synthetic biology techniques can deliver therapeutic drugs to the liver or gastrointestinal tract [7], demonstrating certain advantages in enhancing local drug concentration and reducing systemic adverse reactions.

**Fig. 1:**
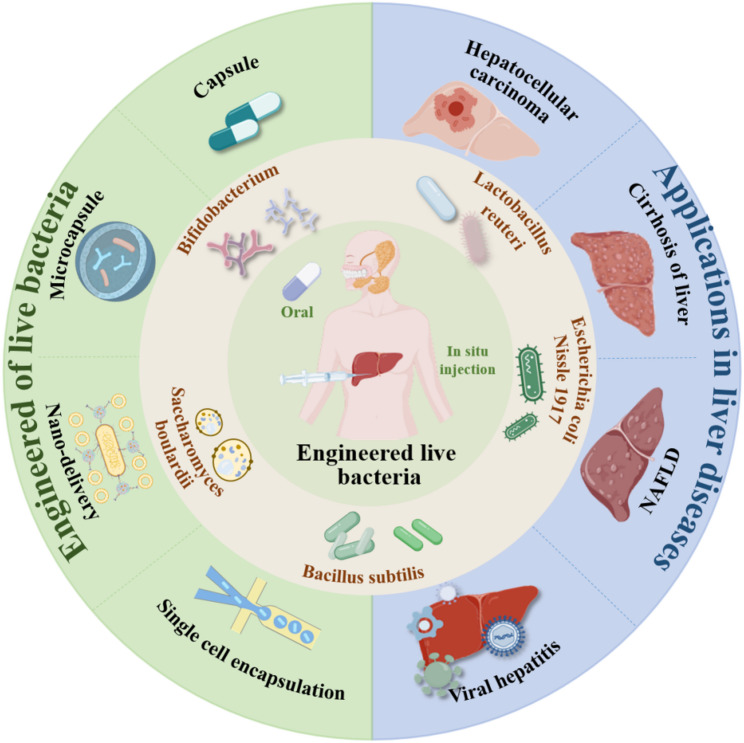
Engineered live bacteria and their treatment of liver disease and gut-liver axis related disorders (created by BioRender.com)

This article reviews the research on the application of engineered live bacteria in the treatment of liver diseases. Firstly, we discuss several original live bacteria used in treating liver diseases, as well as the related engineering modification strategies, focusing on their mechanism of action and therapeutic applications in liver diseases. It also pays attention to the internal genetic modification and external covalent connection of the original live bacteria. Secondly, we also study formulation design and delivery strategies, including capsule formulations, microcapsules, nanoscale delivery systems, and single-cell capsules. Finally, we discuss the challenges and future prospects of using engineered live bacteria to treat liver diseases and diseases related to the gut-liver axis, emphasizing the potential of these innovative treatment methods in reducing the global burden of liver diseases (Fig. 1). Through the combined application of genetic engineering, synthetic biology, and nanotechnology, we aim to clarify the optimization strategies of engineered live bacteria. We aim to find effective and targeted treatment strategies to provide better therapeutic effects and a better quality of life for patients with liver diseases.

## Emerging live bacteria

The liver is vital for orchestrating essential bodily processes, from metabolism and detoxification to protein synthesis and immune regulation [8, 9]. Hepatic pathologies involve a defined sequence of hepatocellular damage and inflammatory influx, leading to subsequent activation of hepatic stellate cells, which results in progressive impairment of liver function and disruption of hepatic architecture [10]. Approximately 2 million deaths annually are attributed to liver diseases, representing 4% of global mortality. There are many clinical strategies for treating liver diseases, such as surgical treatment, drug therapy, and liver transplantation. Although these treatment methods and traditional medicines have achieved certain therapeutic effects, they still face many challenges. For example, the raw materials of traditional synthetic drugs are mostly chemical reagents or rare natural products that rely on non-renewable resources; the biological barriers in the liver reduce the amounts of drugs that enter the body circulation, inhibiting the therapeutic effect of the drugs [11, 12]. Fortunately, natural drugs, especially live bacterial drugs, due to their natural and degradable nature, are easier to obtain.

Currently, the clinical management of liver diseases relies primarily on surgical interventions and pharmacotherapy [13]. Natural therapeutics, particularly live bacterial agents, have garnered growing interest among researchers. Compared with conventional synthetic drugs, these biologic agents offer multiple advantages. For instance, synthetic drugs are often derived from chemical reagents or scarce natural products dependent on non-renewable resources, whereas live bacterial drugs are predominantly sourced from readily available, biodegradable natural material [14, 15]. Furthermore, live bacterial agents are produced through biotechnological fermentation, which streamlines the manufacturing process, facilitates scalability, and supports sustainable and cost-effective drug production. Among the live bacteria applicable for liver disease treatment. Intestinal barrier integrity is influenced by Gram-positive strains, with species like *Bifidobacterium*, *L. reuteri*, and *B. subtilis* playing a role. Gram-positive bacteria like *Bifidobacterium* exert effects via bile acid metabolism and immune modulation, while yeast strains such as *S. boulardii* primarily mediate systemic anti-inflammatory responses [16, 17].

This review focuses on several representative live bacterial agents, including *Bifidobacterium*, *B. subtilis*,* S. boulardii*, *L. reuteri*, and EcN (Table 1). We confirmed that these active biological preparations achieve multi-level intervention by regulating the intestinal microbiota, metabolites, and the network of immune axes. It is worth noting that these active bacteria enhance the natural barrier functions of the intestine, liver, and other organs, thus preserving the homeostasis of biological systems during the treatment of liver diseases.

**Table 1:** Living bacteria for liver diseases and gut-liver axis diseases

Live bacteria	Sources	Advantages	Applications	References
*Bifidobacterium*	-Isolated and screened from the intestines of healthy humans or human milk -Isolated from traditional fermented dairy products	-Strong intestinal mucosa affinity, easy to colonize and form stable flora -High safety	-Inflammatory bowel disease (IBD) -Irritable bowel syndrome	[18, 19]
EcN	-Isolated from the feces of healthy humans -Produced through artificial directed cultivation	-High safety -Strong ability to resist gastric acid and bile -Inhibit the proliferation of harmful intestinal microorganisms	-IBD -Metabolic disorders -Infectious diarrhea	[20, 21]
*B. subtilis*	-Isolated from natural environments such as soil and the rhizosphere of plants -Isolated from traditional fermented dairy products	-Clear genetic background -Strong protein secretion ability -Probiotic characteristics	-Diseases of the digestive system -Dysbiosis of the intestinal flora	[22–24]
*S. boulardii*	-Isolated from tropical fruit peels -Artificial pure culture production	-Good environmental adaptability -Biological barrier function -Anti-inflammatory and immune regulation	-Diarrhea-related diseases -Auxiliary treatment of liver diseases	[25, 26]
*L. reuteri*	-Isolated and screened from the intestines of healthy humans or human milk -Isolated from traditional fermented dairy products	-Strong intestinal colonization ability -Multi-pathway regulation of immunity	-Gut mucosal inflammation -Immune regulation and anti-infection -Colorectal cancer (CRC)	[27, 28, 29]

### *Bifidobacterium*

Members of the phylum *Actinobacteria*, *Bifidobacterium* species are Gram-positive, anaerobic, non-motile, and non-spore-forming bacteria that exhibit a pleomorphic, rod-shaped morphology. These bacteria display morphological diversity, presenting as curved rods, short rods, or branched Y-shaped forms [30]. Phylogenetically, the genus is organized into ten distinct clusters and exhibits broad host adaptability within the mammalian gastrointestinal tract. To date, approximately 80 species have been identified, and *Bifidobacterium* ranks among the dominant bacterial groups within the intestinal ecosystem of healthy infants [31]. *Bifidobacterium* plays a role in promoting human health, demonstrating potential antidepressant, anxiolytic, and anti-nociceptive effects in patients with irritable bowel syndrome [32].

Emerging evidence highlights the significant role of *Bifidobacterium* in liver and intestinal disorders. *Bifidobacterium* acts as key immunomodulators and promising disease biomarkers, and exerts their biological functions through multiple well-characterized mechanisms [33, 34]. These core mechanisms include the synthesis of short-chain fatty acids (SCFAs) and vitamins, the promotion of immune system maturation, and the competitive exclusion of pathogenic microorganisms [35, 36]. As a key beneficial genus within the gut microbiota, *Bifidobacterium* contributes to gut homeostasis through the secretion of SCFAs and suppression of pathogenic bacterial proliferation [37, 38]. *Bifidobacterium* can also effectively prevent the translocation of bacterial derivatives, such as lipopolysaccharide, to the liver via the portal vein, thereby alleviating hepatic inflammatory responses and endoplasmic reticulum stress [39]. Furthermore, its adherence to the intestinal mucosa facilitates the regulation of permeability to microbes, nutrients, and metabolites, while the biosynthesis of antimicrobial agents by *Bifidobacterium* inhibits the growth of enteric pathogens [40, 41]. It reduces the production of potentially hepatotoxic metabolites, such as amines and phenols, at the source, thereby achieving indirect liver protection. It is worth noting that *Bifidobacterium* has shown antitumor activity in vivo, mediated by the elimination of carcinogens, modulation of the local pH, and induction of protective antimutagenic responses [42].

*Bifidobacterium* has been shown to inhibit HCC development in animal models [43, 44]. Specifically, it displays certain immunomodulatory and anti-inflammatory properties, including the promotion of Foxp3 + regulatory T cells, strengthening the structural integrity of the gut mucosal defense system, and suppression of pro-inflammatory Th2 and Th17 pathways [45]. A study further revealed that *Bifidobacterium* activates the hepatic farnesoid X receptor (FXR) pathway, thereby downregulating lipogenic gene expression and reducing endotoxin translocation to the liver, suggesting an emerging direction for metabolic dysfunction-associated steatotic liver disease management. As a nuclear receptor activated by bile acids, FXR supports gut barrier function in the gut and modulates lipid metabolism in the liver [46, 47]. Following *Bifidobacterium* supplementation in mice, both hepatic steatosis and insulin resistance showed improved [48]. In summary, *Bifidobacterium* may help delay the progression of liver diseases related to steatosis and insulin resistance by enhancing the intestinal barrier, inhibiting pro-inflammatory pathways, and activating the FXR receptor.

### *Escherichia coli Nissle 1917* (EcN)

EcN is a Gram-negative, viable bacterial strain known for its ability to colonize the gastrointestinal tract. Compared to *Bifidobacterium* and other intestinal microorganisms, EcN has a stronger competitive growth advantage and exhibits antagonistic effects against pathogenic *Enterobacteria* [49]. Historically, EcN has been used for the prevention and therapy of gastrointestinal disorders and immune-related conditions. In both humans and livestock, EcN has shown efficacy in reducing diarrhea through mechanisms including immune modulation and competitive exclusion of pathogens [50]. Commercially available as Mutaflor^®^, EcN is used as a probiotic for adults and infants to combat intestinal infections and a range of IBD [51]. During recent years, the scope of EcN applications has broadened, building on its established history of safe use in humans. Notably, EcN has become a key platform for genetic engineering to develop engineered live bacteria for treating CRC and IBD, and metabolic disorders.

EcN contributes to host defense by strengthening the structural integrity of the intestinal epithelial barrier and regulating immune function. Specifically, EcN enhances intestinal barrier integrity by increasing the production of core tight junction proteins, including occludin and ZO-1 [52]. Furthermore, EcN signals through TLR-2 and TLR-4 dependent mechanisms to engage host immune responses, regulating the secretion of various immune mediators and promoting immune homeostasis [53]. The probiotic efficacy of EcN is also attributed to its ability to colonize the gastrointestinal tract, where it exhibits a competitive growth advantage and antagonizes pathogenic *Enterobacteria*. This colonization capacity underpins its therapeutic use in managing intestinal infections and inflammatory conditions in both adults and infants [54, 55]. For instance, EcN strengthens the antibacterial response of human colonocytes and confers protection against *Campylobacter* infection by inducing anti-inflammatory cytokine secretion and activating the anti-apoptotic Akt signaling pathway.

Beyond infectious and inflammatory contexts, EcN has attracted interest for its potential in tumor therapy. As an anaerobic bacterium, it preferentially colonizes hypoxic and necrotic regions of the tumor microenvironment, making it a candidate vehicle for localized drug delivery [56]. EcN can up-regulate the expression and membrane localization of tight junction proteins in intestinal epithelial cells, contribute to repair the damaged mechanical barrier of the intestinal epithelium, and reduce the translocation of intestinal bacteria, lipopolysaccharides, and other hepatotoxic substances to the liver [57]. Based on this, EcN occupies intestinal epithelial adhesion sites through its robust colonization ability and competitively excludes pathogenic bacteria associated with liver disease. Intestinal dysbiosis is a factor promoting the progression of chronic liver disease. Through the combined action of multiple mechanisms, EcN may support intervention in and protection against chronic liver disease. However, the underlying mechanisms of EcN tumor colonization remain incompletely understood and require further validation in higher-quality studies.

### *Bacillus subtilis* (*B. subtilis)*

Compared with Gram-negative EcN, *B. subtilis*, as a spore-forming Gram-positive probiotic, exhibits greater acid and heat tolerance as well as whole-gut colonization capacity, contributing to intestinal homeostasis and protecting against liver injury. *B. subtilis* is a Gram-positive bacterium commonly found in soil and decomposing organic matter and was among the first organisms to have its genome fully sequenced and annotated [58, 59]. In academic research, the strain *B. subtilis* has been established as a model system for investigating diverse biological processes, including proteomics, protein secretion and transport, cell division, and the development of minimal bacterial cells. However, the application of traditional *B. subtilis* strains as hosts for protein expression and metabolic engineering is often limited by their low natural competence. As a probiotic, *B. subtilis* exhibits a high colonization capacity in the human intestinal tract, reaching both the small and large intestines [60, 61]. *B. subtilis* can promote the growth of beneficial anaerobic bacteria, produce organic acids that lower gut pH, and indirectly suppress harmful microbes [62, 63]. Furthermore, *B. subtilis* enhances host immunity by increasing immunoglobulin and antibody levels, strengthening both cellular and humoral immune responses, and thereby supporting overall immune function. [64, 65].

In medical applications, wild-type *B. subtilis* has been used in the management of various intestinal disorders, including infectious diarrhea, IBD, and gut microbiota dysbiosis [66, 67]. However, the therapeutic efficacy of wild-type strains is often limited and lacks specificity. Through genetic engineering, *B. subtilis* can be endowed with novel functions, such as the targeted delivery of anti-inflammatory molecules, expression of specific antibodies, or the optimization of metabolic pathways, thereby enhancing its treatment potential and range of application. For instance, the strain *B. subtilis* interacts with intestinal stem cells and secretory epithelial cells and activates immune responses via the TLR2-MyD88 signaling pathway. It promotes the secretion of mucin Muc2 by goblet cells and stimulates Paneth cells to release lysozyme and antimicrobial peptides, collectively enhancing the mucosal barrier. Moreover, *B. subtilis* improves the host’s ability to clear *Salmonella* infections and reduces the incidence of bacterial enteritis [68, 69].

In the field of liver diseases, researchers have observed therapeutic effect of *B. subtilis* in a mouse model of acute ethanol-induced liver injury [70]. It attenuates pathological damage such as shortened intestinal villi and loss of intestinal epithelial cells caused by acute ethanol exposure in mice, and supports repair of the intestinal barrier. It also inhibits the excessive activation of the NF-κB/NLRP3 inflammatory pathway in the liver, thereby reducing inflammatory cascade in the liver. In addition, *B. subtilis* modulates the structure of the intestinal microbiota in mice following acute ethanol exposure. It increases the relative abundance of *Bacillus* species in the intestine and contributes to restoration of a healthier microbial community structure.

### *Saccharomyces boulardii* (*S. boulardii*)

Unlike the above-mentioned prokaryotic probiotics, *S. boulardii*, as a non-pathogenic probiotic yeast, exhibits greater tolerance to gastric acid and bile, and shows promise in modulating intestinal microecology and supporting management of chronic liver diseases. *S. boulardii* is a non-pathogenic probiotic yeast with demonstrated efficacy in various gastrointestinal disorders. It has now become the most studied and widely applied strain of baker’s yeast *S. boulardii*. As a yeast, *S. boulardii* has heat-resistant and acid-resistant properties, conferring distinct biological advantages. First, its facultative anaerobic supports rapid proliferation in the intestinal tract, robust tolerance to gastric acid and bile, and production of bioactive substances such as antimicrobial peptides, B vitamins, and organic acids [71, 72]. Second, *S. boulardii* has a favorable safety profile, with a low incidence of adverse reactions, mostly being mild gastrointestinal symptoms such as transient bloating or diarrhea [71]. Based on these well-characterized biological and probiotic functions, *S. boulardii* has emerged as a focus of investigation for liver disease interventions.

*S. boulardii* can modulate the composition of the intestinal microbiota through various pathways, thereby supporting intestinal microbial homeostasis in individuals with liver disease. Research findings indicate that *S. boulardii* exerts an inhibitory effect on the proliferation of opportunistic pathogens such as EcN and *Enterococcus* species, while promoting the growth of beneficial bacteria such as *Bifidobacterium* species and *Lactobacillus* species [73, 74]. During the course of liver diseases, inflammatory response and oxidative stress are key pathological mechanisms, and *S. boulardii* can exhibit anti-inflammatory and antioxidant properties through various cellular pathways, potentially mitigating liver injury. For example, in the context of anti-inflammatory actions, *S. boulardii* exerts an inhibitory effect on the generation and secretion of a panel of inflammatory cytokines, notably TNF-α, IL-6, IL-1β, and IL-18 [75–77].

A clinical study enrolling individuals with decompensated cirrhosis showed that *S. boulardii* supplementation was associated with decreased serum levels of C-reactive protein and IL-18, along with attenuation of systemic inflammatory responses [78, 79]. In animal models of metabolic dysfunction-associated steatotic liver disease, it has been shown that *S. boulardii* intervention can reduce hepatic inflammation, downregulate the expression of inflammatory cytokines such as TNF-α and IL-1β, and alleviate the degree of hepatic steatosis and fibrosis [26]. From a mechanism perspective, *S. boulardii* may suppress NF-κB-mediated signaling, thereby attenuating the transcriptional activation and production of inflammatory cytokines [80]. When evaluating antioxidant activity, *S. boulardii* can enhance the catalytic activity of major antioxidant enzymes, notably superoxide dismutase, glutathione peroxidase, and catalase in liver tissue, and reduce the accumulation of oxidative stress products such as malondialdehyde and protein carbonyls. In a mouse model of diabetes-associated liver injury, *S. boulardii* intervention reduced the concentration of protein carbonyls in liver tissue, increased the activities of superoxide dismutase and glutathione peroxidase, and alleviated hepatic oxidative stress damage in this model [81].

### *Lactobacillus reuteri* (*L. reuteri*)

*L. reuteri* is a Gram-positive, catalase-negative, facultatively anaerobic, rod-shaped bacterium belonging to the phylum Actinomycetota and the order *Lactobacillales*. Initially identified in the early 20th century as a biotype of *Lactobacillus fermentum*, it colonizes diverse niches, including the human and animal gastrointestinal tracts, oral cavity, and breast milk. In the 1990 s, research revealed that *L. reuteri* synthesizes reuterin, a broad-spectrum antimicrobial compound active against many Gram-positive bacteria and some viruses, suggesting potential utility in managing infectious diseases [82].

As research into the gut-liver axis advances, the potential role of *L. reuteri* in managing liver diseases has attracted increasing attention. Increasing evidence indicates that this bacterium has beneficial effects on various liver diseases through multiple mechanisms, including regulating the intestinal microbiota, fortifying the intestinal barrier, regulating immune activity, and modulating lipid metabolism. In this regard, *L. reuteri* regulates immune function through multiple pathways to alleviate liver inflammation [83, 84]. For example, it increases the quantity and functional capacity of regulatory T cells, while inhibiting excessive Th1 and Th2 activation, thereby reducing immune-mediated liver damage [85]. At the cellular level, *L. reuteri* promotes the acetate-GPR43 signaling pathway, which orchestrates functional repolarization of macrophages, shifting their phenotype from the pro-inflammatory M1 state toward an anti-inflammatory state [86, 87]. Additionally, it suppresses elevated expression of certain pro-inflammatory cytokines such as TNF-α and concurrently enhances secretion of the anti-inflammatory cytokine IL-10 [84, 88]. Collectively, these immune regulatory effects help to alleviate liver inflammation and slow down disease progression.

Oxidative stress is another key contributor to liver pathogenesis. Notably, *L. reuteri* exhibits certain antioxidant activity, which helps mitigate oxidative injury in the liver. Research findings suggest that it reinforces the activity of major antioxidant enzymes while reducing levels of lipid peroxidation byproducts particularly malondialdehyde [89]. Moreover, *L. reuteri* may have therapeutic effects in diseases related to the gut-liver axis, particularly those involving intestinal barrier dysfunction. It promotes the secretion of IL-22, a cytokine critical for mucosal repair. Specifically, its metabolite indole-3-aldehyde activates lamina propria lymphocytes via the aryl hydrocarbon receptor, stimulating IL-22 release. This, in turn, induces phosphorylation of STAT3, enhancing the proliferation of intestinal lining cells and supporting regeneration of damaged intestinal mucosa [90].

While the five probiotic species discussed above share common beneficial properties, their mechanisms of action exhibit both convergent and divergent features that critically influence their suitability for specific liver disease applications and engineering strategies. All five species converge on three fundamental protective pathways relevant to liver diseases: (i) intestinal barrier reinforcement, (ii) immune modulation, and (iii) metabolite production. All species upregulate tight junction proteins (Occludin, ZO-1, Claudins) to reduce endotoxin translocation to the liver, though *Bifidobacterium* and *L. reuteri* demonstrate superior mucosal adhesion capacity. Immune modulation represents another shared feature: EcN and *B. subtilis* activate TLR2/4 signaling, while *Bifidobacterium* and *L. reuteri* preferentially promote regulatory T-cell responses. SCFA production (acetate, butyrate, propionate) is universal but quantitatively variable, engineered *B. subtilis* SCK6 achieves the highest butyrate titers, whereas *S. boulardii* primarily produces other organic acids.

Besides, EcN is optimal for tumor-targeted delivery to hypoxic HCC lesions due to its facultative anaerobic and hypoxia-inducible genetic circuits. *B. subtilis* is preferred for protein-based therapeutics (e.g., antibodies, enzymes) requiring high-yield secretion. *Bifidobacterium* and *L. reuteri* represent the safest options for chronic liver diseases (NAFLD, cirrhosis) due to their GRAS status, lack of endotoxin, and superior mucosal colonization. *S. boulardii* offers unique advantages for post-translational modification of complex proteins but requires careful consideration of fungal infection risks in immunocompromised patients. Critically, these species are not mutually exclusive alternatives but potentially complementary components of therapeutic regimens. For instance, EcN’s tumor-targeting capacity could be combined with *B. subtilis*-derived therapeutic proteins, or *Bifidobacterium*-mediated barrier repair could precede EcN-based targeted therapy. Understanding these mechanistic relationships enables rational design of multispecies engineered therapeutics that exploit complementary rather than redundant functions.

## Engineered strategies of live bacteria

Live bacteria often encounter numerous challenges during the therapeutic application. For instance, excessive metabolic burden can reduce therapeutic efficacy, and the loss of surface functions increases their susceptibility to immune recognition and clearance [91]. To overcome these limitations, researchers are exploring multiple dimensions to precisely reprogram living bacteria using engineering methods. First, genetic modification can be used to engineer living bacteria. This mainly involves knocking out virulence genes or integrating exogenous functional modules to give them stable drug synthesis and regulation capabilities. Second, covalent surface modification is also a common method for modifying bacteria. By using bioorthogonal reactions or amide condensation, antibodies or nanoparticles can be coupled to the bacterial surface, thereby transforming them into active biological interfaces that can recognize specific lesions. Third, structural design can be used to engineer living bacteria. This mainly involves constructing physical barriers in the form of microcapsules or hydrogel matrices to isolate the acidity of the gastrointestinal tract or host immune surveillance. By systematically refining these bacterial engineering strategies, a robust platform of engineered live bacterial therapeutics can be developed for disease treatment.

### Genetic modification

Gene editing technology involves integrating specific genetic circuits into the bacterial genome, and it possesses capabilities such as precise targeting, dynamic response, efficient delivery, and immune regulation [92, 93]. Targeted gene modification can reduce the risk of bacteria being rapidly cleared by the host’s immune system [94]. In addition, bacteria that have been genetically edited can reduce the immune system’s attack on them, thereby achieving a longer survival period and more stable therapeutic functions within the host. This modification can enhance the inherent advantages of bacteria in tumor-targeted therapy, enabling them to actively move towards the tumor microenvironment and work synergistically with other anti-cancer drugs [95]. Currently, the gene editing strategies for live bacteria mainly include CRISPR/Cas9 technology, zinc finger nuclease (ZFN) technology, and transcription activator-like effector nuclease (TALEN) technology. This study aims to clarify the intrinsic mechanism of action of these technologies and explore the potential value of their technical characteristics in the application of engineered bacteria to the field of liver diseases.

#### CRISPR/Cas9 technology

The CRISPR/Cas9 system is a specialized defense mechanism in prokaryotes, which can resist foreign genetic elements such as bacteriophages and plasmids. This system comprises CRISPR arrays and a suite of Cas proteins. Within this framework, guided by a CRISPR RNA in complex with a trans-activating crRNA, Cas nucleases recognize and cleave invading nucleic acids from viruses and plasmids in a sequence-specific manner. When engineered for genome editing, the CRISPR/Cas9 system employs a sgRNA to direct the Cas9 endonuclease to induce double-strand breaks at defined genomic loci via Watson-Crick base pairing between the sgRNA spacer and the target DNA sequence. Upon binding to the target sequence, sgRNA recruits Cas9 protein, which induces a double-strand break in the DNA [96, 97]. It is worth noting that CRISPR/Cas9 has many advantages over traditional gene editing methods. For example, relocating to a new gene locus only requires designing specific sgRNA, while the Cas9 protein remains unchanged in various applications, providing convenience for researchers. Additionally, co-expressing Cas9 protein with multiple guide RNAs can simultaneously edit multiple targets within the mammalian genome. Therefore, this technology is widely used by researchers due to its simple design, high efficiency, and low cost-effectiveness [96, 98].

**Fig. 2:**
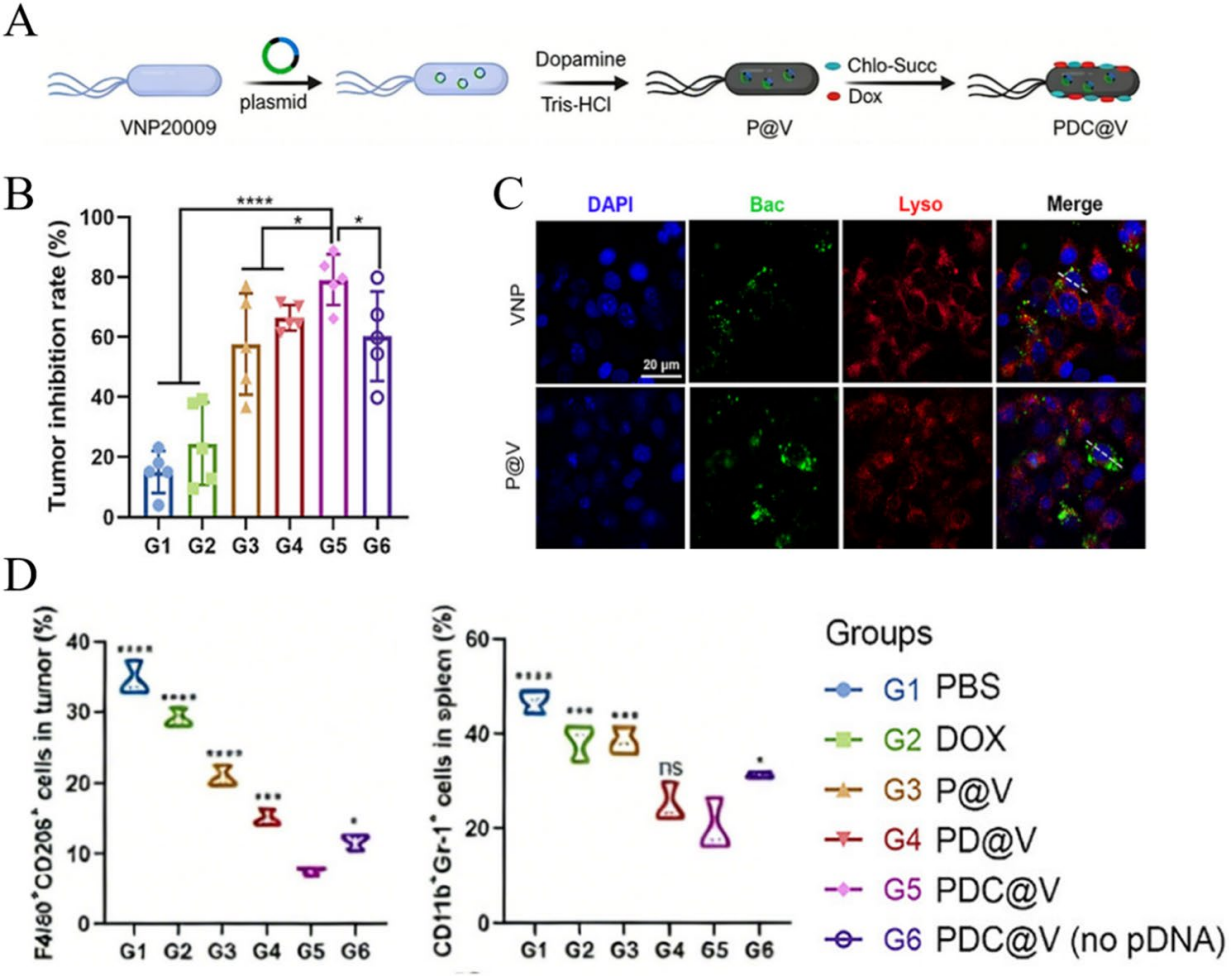
(**A**) The schematic diagram of the modification of PDC@V made by gene knockout and functional gene introduction of VNP20009. (**B**) Tumor growth inhibition rates in 4T1 tumor-bearing mice after different treatments. (**C**) CLSM images of endo-/lysosomal escape of engineered bacteria in 4T1 cells. Scale bar, 20 μm. (**D**) Quantitative analysis of immunosuppressive M2-type macrophages and myeloid-derived suppressor cells in the tumor tissues of 4T1 tumor-bearing mice. Copyright^©^2025, Advanced Functional Materials

Researchers have confirmed that *Salmonella* can be engineered using the CRISPR/Cas9 system. An et al. conducted dual genetic modifications on the attenuated *Salmonella typhimurium* VNP20009 (VNP20009), including gene knockout and the introduction of functional genes, resulting in the engineered strain PDC@V [99]. Firstly, it targeted and knocked out the *Slc7a2* gene to enhance the safety of the strain for application. After completing the gene knockout, they introduced two types of core functional elements into the strain, namely, listeriolysin O and the CRISPR/Cas9 system targeting the murine *Slc7a2* gene (Fig. 2A). The expression of listeriolysin O was driven by a hypoxia-inducible promoter. In tumor models, this engineered bacterium can overcome the resistance of tumor cells to doxorubicin. Under hypoxia induction, PDC@V expresses the listeriolysin O protein, facilitating bacterial escape from endosomes into the cytosol (Fig. 2B-C). The results show that PDC@V can reverse the polarization of macrophages in the tumor microenvironment from the M2 type to the M1 type (Fig. 2D). Additionally, the released CRISPR/Cas9 system edits the *Slc7a2* gene of tumor cells, reducing the expression of this arginine transporter and thereby blocking the uptake of exogenous arginine by tumor cells, reversing their chemotherapy resistance.

*Bifidobacterium* species can also undergo genetic modification through the CRISPR/Cas9 system. The researchers successfully constructed the cBEST gene editing system by knocking out three restriction endonuclease genes in *Bifidobacterium* [100]. This gene editing system circumvents the host’s restriction-modification barrier, thereby enhancing both plasmid transformation efficiency and genome editing fidelity. For large plasmids containing the HsdR recognition sequence, the transformation efficiency was also increased by approximately 300,000-fold, reaching 10^6^−10^7^ CFU/µg. Additionally, the researchers combined exogenous CRISPR/Cas9 counter-selection with endogenous RecT-mediated single-stranded DNA recombination to construct a gene editing system in *L. reuteri* [101]. This system achieved 90% to 100% efficiency in precise point mutations, knockout, deletion, and codon saturation mutations of genes such as *lacL*, *srtA*, and *sdp6*, solving the problems of low recombination efficiency and difficult screening in *L. reuteri.* Therefore, through CRISPR/Cas9 technology to modify natural live bacteria to intervene in intestinal inflammation and delay tumor progression has achieved significant progress, which will provide new ideas and theoretical basis for exploring treatment strategies for liver diseases.

#### Zinc finger nuclease (ZFN) technology

ZFN is the first version of gene editing technology, marking a new era of targeted genome modification [102]. It is a modular structure composed of a zinc finger protein DNA-binding domain fused to the FokI restriction endonuclease cleavage domain. The zinc finger protein can specifically recognize and bind to specific DNA sequences. Compared with the CRISPR/Cas9 system, its functions are entirely achieved by proteins without the introduction of any exogenous RNA. Therefore, the ZFN system has an advantage in terms of immunogenicity control and host cell adaptability.

**Fig. 3:**
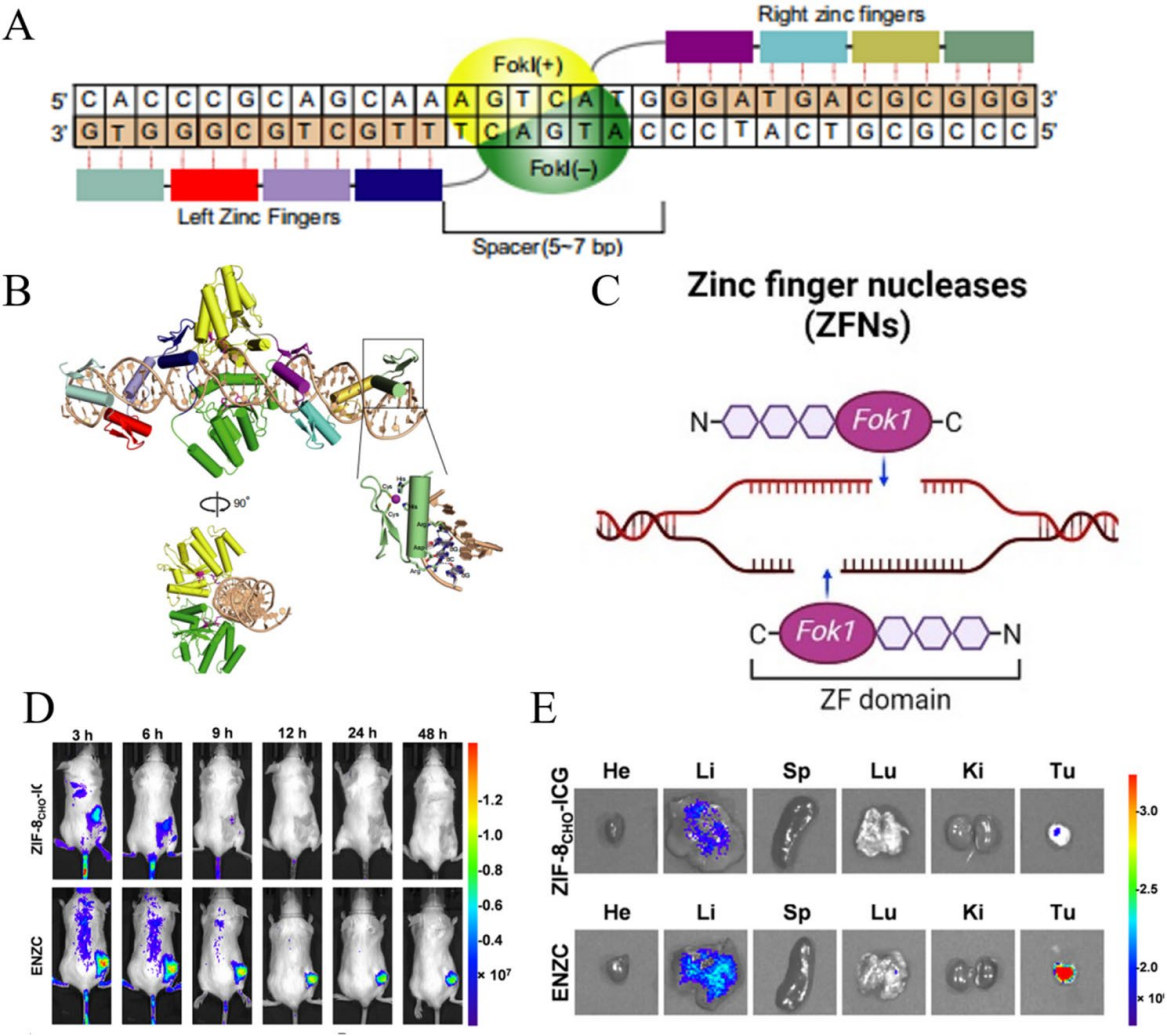
(**A**-**B**) ZFN architecture. Corresponding domains maintain consistent coloring across both (**A**) and (**B**) panels. Copyright^©^2016, Plant Biotechnol J. (**C**) ZFN generate double-strand DNA breaks. Copyright^©^2024, Mol Ther. (**D**) The tumor condition in mice after intravenous injection of ZIF-8CHO-ICG. (**E**) In vitro imaging of the tumor in mice after intravenous injection of ZIF-8CHO-ICG. Copyright^©^2024, ACS Nano

The zinc finger protein array consisting of multiple tandemly linked zinc motifs can recognize specific 9–12 base sequences in the genome. The FokI restriction endonuclease is responsible for the cutting function [103, 104]. When ZFN binds to the target gene, FokI will cut the DNA to form a double-strand break, thereby triggering the cell’s repair mechanism and achieving gene editing (Fig. 3A-C). In research on liver diseases, ZFN has demonstrated its unique advantages [105]. For instance, researchers have used this technology to modify EcN, enabling it to co-express anti-PD-L1 and anti-CD9 nanobodies (EcN-Nb). By loading indocyanine green (ICG) onto zinc-based metal-organic frameworks to modify EcN-Nb, they prepared EcN-Nb-ZIF-8CHO-ICG (ENZC) for immunotherapy. In vivo fluorescence imaging of mouse models showed that the tumor accumulation of ENZC was significantly stronger than that of the control group (Fig. 3D-E). Additionally, ZFN technology can be combined with lipid nanoparticles for delivery, allowing for repeated dosing in patients and not being limited by pre-existing neutralizing antibodies in the patient’s body. However, because natural live bacteria lack canonical non-homologous end joining machinery, ZFN-induced DSBs frequently trigger bacterial cell death, severely impeding precise, scarless gene editing in prokaryotic hosts [106]. Therefore, this limitation still requires further technological optimization in the future.

#### Transcription activator-like effector nuclease (TALEN) technology

TALEN technology originated from the study of plant pathogens, especially bacteria from *Xanthomonas* species. Upon infecting plants, these pathogens utilize the type III secretion system to enable the functional delivery of transcription activator-like proteins to host cells for genetic manipulation [107]. These effectors specifically bind promoter regions in the plant genome and activate susceptibility gene expression, thereby facilitating bacterial colonization and propagation. TALEN technology consists of a customizable transcription activator-like effector DNA-binding domain fused to the FokI nuclease cleavage domain. A strict one-to-one correspondence exists between the amino acid residues at positions 12 and 13 of each transcription activator-like effector repeat module and its cognate DNA base, enabling programmable, sequence-specific DNA recognition. By designing different transcription activator-like protein structures, it can specifically recognize and bind to different DNA sequences. It is worth noting that TALEN technology achieved a pivotal breakthrough in 2009 [108, 109]. A research team revealed the molecular code of transcription activator-like protein to recognize DNA, and found that the repeated amino acid sequence module in each transcription activator-like protein could specifically recognize the corresponding DNA bases [110]. Moreover, the amino acid residues at positions 12 and 13 in each repeat module determine the specificity of the recognition. Similar to ZFN, TALEN also fuses transcription activator-like protein with FokI restriction endonuclease. When a TALEN pair binds to adjacent, inversely oriented target DNA sequences, dimerized FokI domains induce a site-specific double-strand break, thereby activating endogenous cellular DNA repair pathways, including non-homologous end joining and homology-directed repair, to enable precise genome editing in engineered live bacterial strains [108]. For example, knockout of virulence-related genes in some native live bacteria reduces their potential pathogenicity while retaining their beneficial functions [111].

Currently, TALEN technology has been applied in research related to live bacteria modification. Some researchers used TALEN technology to simultaneously knock out the *FAA1* and *FAA4* genes in *Saccharomyces cerevisiae*, blocking the recycling and utilization of free fatty acids and significantly increasing the accumulation of intracellular fatty acids in yeast [112]. The research results showed that about 9.4% of the cells in the modified strain had obvious lipid droplets, and the total fatty acid production reached 10.28 µg/10^8^ cells, which was higher than that of the control. This strategy proved the application value of TALEN in multi-gene editing of industrial microorganisms. Other researchers used TALEN technology in combination with overexpression of the exonuclease RoKem1 to successfully achieve targeted knockout of the *trpC* gene in the *Mucorales fungus Rhizopus oryzae* [113], obtaining a tryptophan auxotrophic mutant strain. The results indicated that this fungus mainly relied on microhomology-mediated end joining to repair DNA double-strand breaks. This method provided the first TALEN gene editing tool for *Mucorales fungus*, which can be used for metabolic engineering modification of industrial strains and research on the pathogenic mechanism of pathogenic fungi. Moreover, researchers fused TALEN to the *Pseudomonas aeruginosa* type III secretion system effector ExoS54 signal peptide and a nuclear localization signal, enabling direct cytosolic delivery and subsequent nuclear translocation of functional TALEN protein into human HeLa cells via bacterial injection, achieving targeted knockout of the *Venus* reporter gene [114]. This method not only ensured an editing efficiency of over 20%, but also had higher safety due to the absence of exogenous gene integration. Researchers also used TALEN technology to knock out the *adp* gene in the nematode pathogen *Bacillus nematocida* B16 [111]. The study found that the *adp* gene plays a key role in the colonization of the nematode intestine by the *Bacillus nematocida* B16 strain and in its insecticidal activity. All these provide evidence that TALEN technology has been applied in research related to live bacteria modification. However, TALEN technology has problems such as high cost and cumbersome module assembly, making it difficult to conduct large-scale research in live bacteria modification.

### Covalent connection

Although gene modification can achieve precise programming of bacterial metabolic and synthetic pathways at the genetic level, it is difficult to endow it with non-genetic encoded dynamic interface characteristics. However, covalent connection serves as an important supplementary means for bacterial external editing and compensates for this limitation. Covalent bonds, defined by shared electron pairs between atoms, constitute the strongest class of chemical interactions and underpin stable surface modifications of bacterial cells [115]. They can also be involved in the modification of engineered live bacteria after gene editing, enhancing the stability and therapeutic effect of the engineered live bacteria. Compared with traditional physical methods, this covalent bond strategy (including bioorthogonal reactions and amide condensation reactions) has higher stability and wide applicability in various materials such as amino acids, nanozymes, metal nanoparticles, and nanomedicines. In the external modification of bacteria, the chemical coupling strategy based on covalent bonds can stably and irreversibly link functional molecules to the bacterial surface through specific chemical reactions [116].

#### Bioorthogonal reactions

Bioorthogonal reactions are a type of chemical transformation that can occur within living systems without interfering with the original biological processes [117]. These reactions have the characteristics of rapid reaction kinetics, high efficiency, mild conditions, and excellent biocompatibility. They can label and functionalize the surface of bacteria without damaging the activity and integrity of the living bacteria. According to different reaction mechanisms, the representative bioorthogonal reactions that have been developed include Staudinger ligation, copper-catalyzed azide-alkyne cycloaddition, and inverse electron-demand Diels-Alder reactions. In recent years, researchers have expanded bioorthogonal chemistry to develop advanced “click-to-release” strategies [118]. By triggering specific chemical bond cleavage through bioorthogonal reactions, they can achieve the controlled release of functional molecules, providing new tools for constructing engineered living bacteria systems with precise spatiotemporal regulation.

**Fig. 4:**
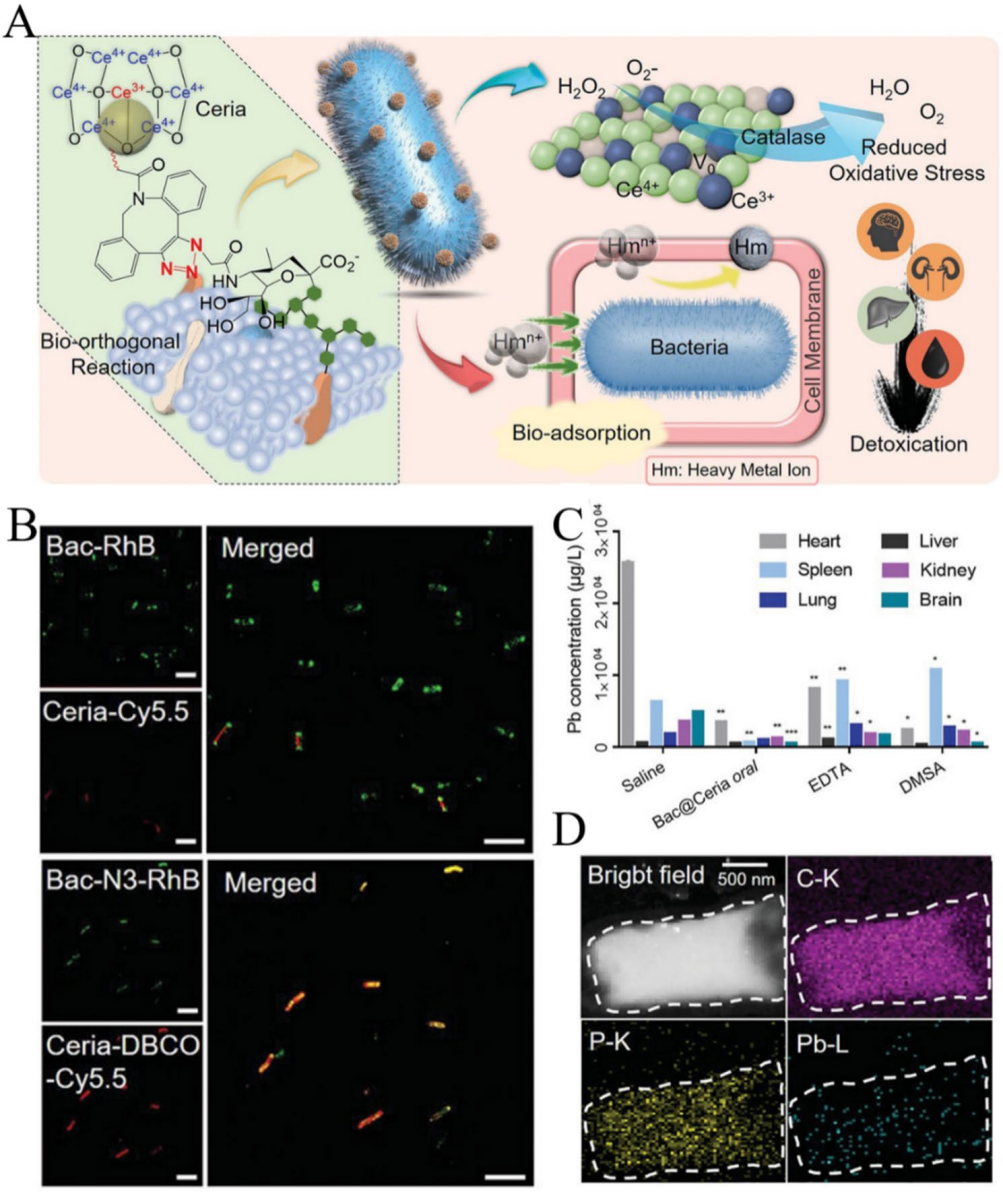
(**A**) Schematic illustration of the bio-orthogonal bacterial reactor and its mechanism for heavy metal detoxification and reactive oxygen species elimination. (**B**) The fluorescence co-localization images illustrate the bioorthogonal conjugation technology of EcN. Scale bar, 20 μm. (**C**) Elemental mapping images of Pb, C, and P distribution on the EcN surface after Pb^2+^ treatment. (**D**) Pb concentrations in major organs of chronic lead-poisoned mice after different treatments. Scale bar, 500 nm. Copyright^©^2025, Adv Sci

Therefore, Pan et al. utilized this more rational click chemical reaction to perform surface functionalization on the non-pathogenic EcN MG1655 [119]. They adopted metabolic glycoengineering combined with copper-free bioorthogonal click chemistry. The bacterial surface was modified with azide groups through 2-azido-2-deoxyglucose metabolic labeling. Subsequently, dibenzocyclooctyne-functionalized cerium oxide nanoparticles underwent strain-promoted azide-alkyne cycloaddition with azide-labeled bacteria, achieving stable, covalent conjugation of cerium oxide to the bacterial cell envelope (Fig. 4A-B). This hybrid system fully integrates the dual advantages of live bacteria and nanomaterials, achieving targeted enrichment and functional synergy in vivo. EcN, relying on its natural bioadsorption and biomineralization, actively accumulates in and is enriched in organs with high lead content such as the liver, kidneys, and spleen, efficiently capturing and eliminating lead ions in the body (Fig. 4C-D). Meanwhile, cerium oxide nanoparticles, through the reversible Ce^3+^/Ce^4+^ cycle, mimicked the activities of superoxide dismutase and catalase, eliminating excessive reactive oxygen species caused by lead poisoning, and alleviating oxidative stress and tissue inflammatory damage.

#### Amide condensation reactions

The bacterial surface is densely covered with abundant protein molecules, and the amino groups in their structures can serve as common targets for chemical modification [117]. Through specific chemical coupling strategies, exogenous functional molecules can be covalently linked to the amino groups on the bacterial surface. Crosslinking agents such as N-hydroxysuccinimide ester and 2-aminosulfonyl chloride have been proven to efficiently react with the amino groups to form stable covalent connections. It is worth noting that the bond dissociation enthalpy is as high as 305–440 kJ·mol^− 1^, which is much higher than that of antibody conjugation systems based on non-covalent affinity interactions [120]. At the same time, this covalent connection exhibits excellent stability in the in vivo environment and is not easily affected by other media. Based on this principle, researchers have successfully anchored functional elements such as activated MnO_2_ nanorings, ZnO nanorods, nanomedicines, and small molecule drugs to the bacterial surface through carbodiimide chemistry.

**Fig. 5:**
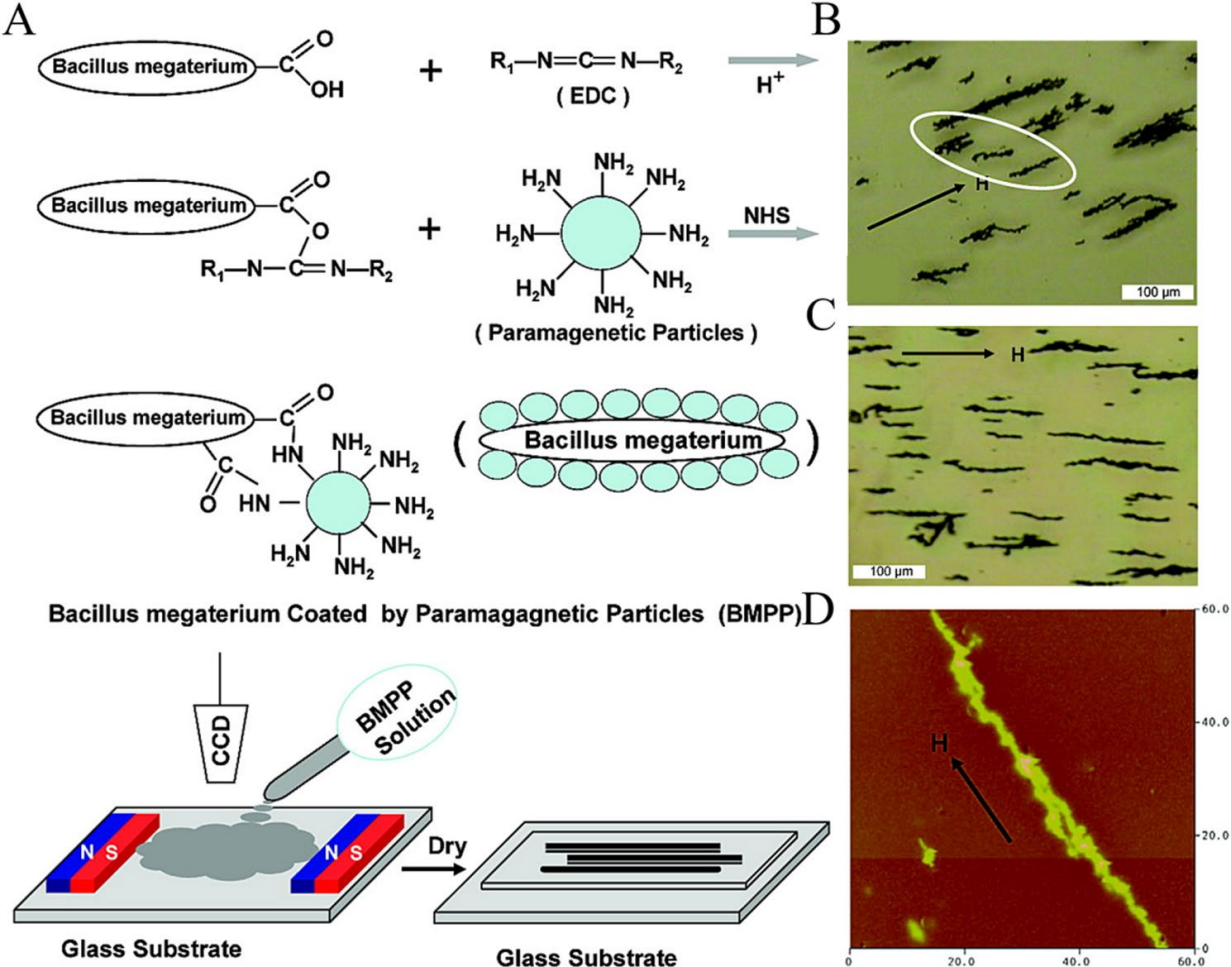
(**A**) Schematic illustration of carboxyl activation and covalent conjugation of magnetic particles on the bacterial surface. (**B**-**C**) Representative images of bacteria modified with magnetic particles. Scale bar, 100 nm. (**D**) Representative topographic images of bacteria modified with magnetic particles. The Z-scale for the 60 × 60 μm. Scale bar, 1000 nm. Copyright^©^2007, Chemistry of Materials

For instance, *Bacillus megaterium* was surface-functionalized via carbodiimide-mediated amidation [121]. First, the carboxyl groups on the bacterial surface were activated by carbodiimide. Subsequently, these activated carboxyl underwent nucleophilic acyl substitution with primary amines on the surface of superparamagnetic nanoparticles, forming stable amide bonds and covalently anchoring the magnetic particles to the bacterial envelope (Fig. 5A). This covalent conjugation firmly immobilizes the nanoparticles on live bacteria (Fig. 5B). The modified live bacteria can be aligned into linear arrays under an external magnetic field and then permanently fixed in a silica matrix through sol-gel polymerization (Fig. 5C-D). This provides a new strategy for the assembly of live bacteria that can be magnetically controlled and have a stable structure for the preparation of microfluidic devices, bioanalysis, and functional materials. This magnetic control technology can be used to construct engineered live bacteria systems, and under the guidance of a magnetic field, it helps live bacteria to accumulate at the lesion sites in the liver.

### Structure of live bacteria

To enable engineered live bacterial therapeutics for clinical translation, in addition to genetic modification and chemical conjugation, researchers have adopted various external approaches to modify the delivery strategies of bacteria. These methods can further enhance the stability, targeting ability, and therapeutic effect of engineered live bacteria in the host. First, to address the damage caused by gastric acid and digestive enzymes such as pepsin during oral delivery, researchers have developed physical barriers for the bacteria through material encapsulation [122]. Currently, multiple protective structures such as conventional capsule encapsulation, microcapsule wrapping, and nano-coating have been studied. These encapsulation methods can increase the survival rate of engineered live bacteria in the gastrointestinal tract, allowing them to safely reach the intestine and stably colonize.

Building on this, researchers have also developed an advanced surface encapsulation technology. Encapsulating individual bacterial cells provides precise protection for engineered bacteria, enabling them to maintain structural integrity and biological activity in complex in vivo environments, offering a promising strategy for liver disease intervention. In addition, the route of administration also has an impact on the treatment of liver diseases. For intestinal diseases related to the gut-liver axis and liver diseases, oral administration is considered the most convenient method due to its simplicity and high patient compliance. For local lesions such as tumors and liver fibrosis, in situ injection strategies can be adopted. For example, intrahepatic injection can precisely deliver drugs to liver parenchymal lesions such as tumors and liver fibrosis under ultrasound/CT guidance [123]. This method can achieve the accumulation of drugs at the lesion site, thereby enhancing the therapeutic effect of liver diseases. In summary, rational design of dosage forms for engineered live bacterial therapeutics must be tailored to both disease phenotype and administration route.

#### Capsule

**Fig. 6:**
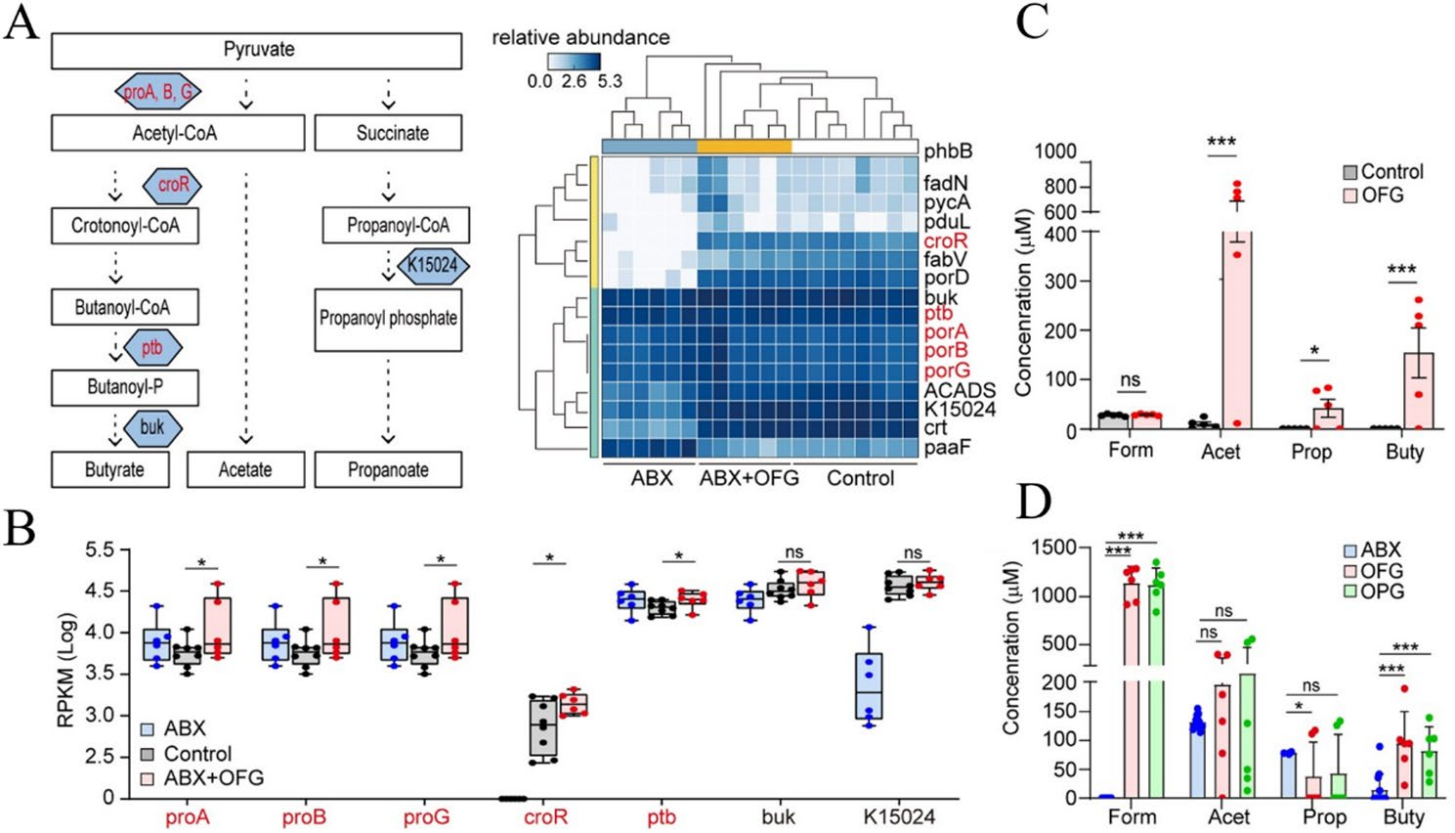
(**A**) Schematic and hierarchical clustering heatmap of key genes involved in bacterial short-chain fatty acid biosynthesis. (**B**) Abundance levels of essential butyrate-producing genes (log-scale RPKM). (**C**) Fecal short-chain fatty acid levels in germ-free mice before (gray bar) and after (red bar) the administration of ABX + OFG. (**D**) Concentrations of SCFAs measured across different fecal suspensions: from ABX mice (gray bar), and following treatment with (OPG, red bar) or without (OFG, green bar) pasteurization. Copyright^©^2024, Cell Rep

Oral capsules are the basis of oral administration of live bacteria preparations and offer the possibility of non-invasive microbiome therapy. The advent of oral capsules has addressed the invasiveness and operational inconvenience of fecal microbiota transplantation performed via colonoscopy or enema, significantly enhancing patient compliance and treatment accessibility. It also plays a role in the treatment of liver diseases [124]. For instance, long-term consumption of live bacteria capsules containing *Lactobacillus* and *Bifidobacterium* can improve the intestinal barrier function of patients with cirrhosis. This effect in turn lowers the risk of endotoxemia, which ultimately alleviates secondary liver inflammation and tissue injury. Researchers have found that oral fecal microbiota transplantation (OFG) capsules have a mitigating effect on acetaminophen-induced acute liver injury [125]. This is because OFG can upregulate the metabolic pathways related to butyrate synthesis and increase the abundance of key genes involved in butyrate synthesis (Fig. 6A-B). The study discovered that the liver-protective effect of OFG does not rely on the colonization of live bacteria but is achieved by increasing the levels of SCFAs such as acetic acid, propionic acid, and butyric acid in the gut, among which butyric acid is the core effector molecule (Fig. 6C-D). This provides a key mechanism for subsequent research on the use of engineered live bacteria to treat liver diseases through the targeted synthesis of butyric acid and other metabolic products. However, such oral capsules require a large dosage to achieve an effective concentration, and the survival rate of live bacteria during the passage through the stomach is relatively low. These issues have greatly restricted the clinical transformation and application of oral live bacterial preparations for engineering purposes. There is still a need to develop oral delivery systems that are resistant to gastric acid, have high delivery efficiency, and require low effective doses.

To address these limitations, researchers have improved the production process and delivery technology. For instance, freeze-drying technology is adopted to concentrate samples and prepare new formulations that are small in volume but high in bacterial content, effectively reducing the number of capsules to be taken at one time [126]. Based on the research of engineered live bacteria capsules, some research teams have developed ingestible optoelectronic devices. Specifically, researchers have engineered EcN to express a nitrate-responsive promoter and a luciferase gene cluster, enabling the bacteria to emit light actively in an inflammatory environment. The capsules capture the light signals through integrated optoelectronic sensors and transmit them to mobile phones, thus achieving early disease detection [127].

Enteric-coated capsules are also an improvement on traditional capsules. The shell material of these capsules has been specially treated to remain insoluble in the acidic environment of the stomach and only dissolve in the alkaline environment of the intestines. Commonly used enteric-coated materials include acrylic resin and cellulose acetate phthalate. This characteristic enables enteric-coated capsules to better protect live bacteria as they pass through the stomach and enhance the release efficiency of live bacteria in the small intestine. In the treatment of liver diseases, live bacteria encapsulated in enteric-coated capsules can effectively regulate the intestinal microecology [128]. For patients with hepatic encephalopathy, the combined use of enteric-coated capsules containing specific bacterial groups can reduce blood ammonia levels [129]. This is because the improvement of intestinal microecology helps to reduce the number of ammonia-producing bacteria in the intestines, lower ammonia production and absorption, and thereby help improve the neurocognitive dysfunction of patients with hepatic encephalopathy. Although capsule technology has laid the foundation for the clinical application of engineered live bacterial preparations, the drug release behavior of capsules is easily affected by individual physiological differences, which limits the realization of more precise treatment strategies.

#### Microcapsule

**Fig. 7:**
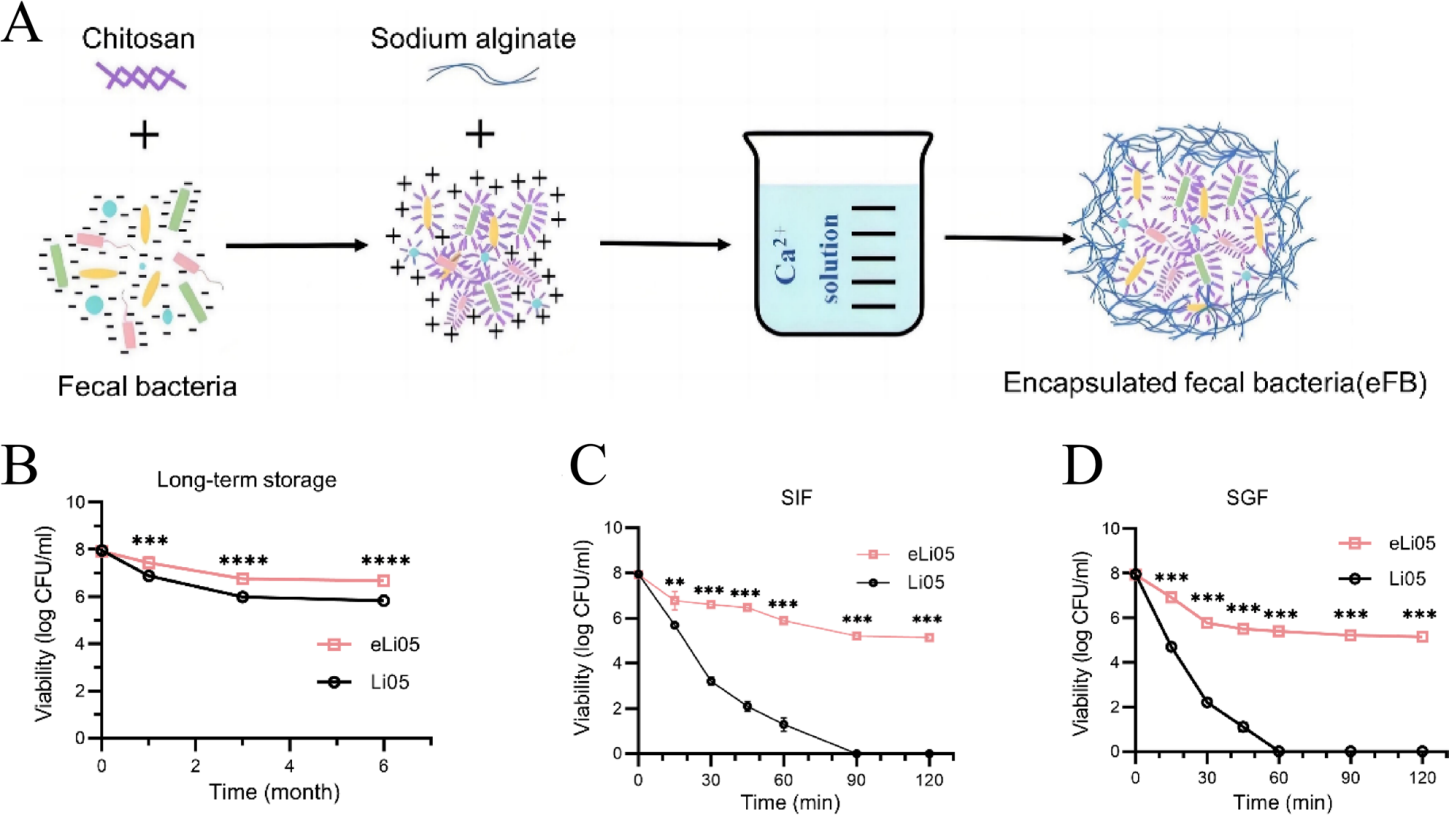
(**A**) Schematic diagram of fecal bacteria embedding principle using microencapsulation method. (**B**-**D**) Effects on viability of Li05 after encapsulation in long-term storage (**B**), SGF (**C**) and SIF (**D**). Copyright^©^2023, Microbiome

Microencapsulation technology involves encapsulating live bacteria in micron-sized protective matrices to ensure their stability during processing, storage, and gastrointestinal transit. Biocompatible and biodegradable materials are widely used in microencapsulation, with polysaccharides and proteins being the main encapsulating materials. A research team utilized the electrostatic self-assembly microencapsulation technique involving chitosan-sodium alginate-Ca^2+^ crosslinking to prepare eFB encapsulating fecal microbiota and eLi05 encapsulating probiotics (Fig. 7A) [130]. The results showed that the long-term storage survival rates of eFB and eLi05 encapsulated by microcapsules were good (Fig. 7B). Moreover, in simulated gastric fluid (SGF) and simulated intestinal fluid (SIF), their survival rates were higher than those of the unencapsulated live bacteria (Fig. 7C-D). This technology aims to construct a physical barrier to effectively isolate external adverse factors such as oxygen, moisture, gastric acid, and digestive enzymes, thereby improving the survival rate of live bacteria. In addition, microcapsules can simultaneously encapsulate living bacteria and cancer chemotherapy drugs to achieve synergistic treatment to enhance efficacy. Microcapsule technology also supports the co-encapsulation of other beneficial substances of living bacteria to further enhance health benefits through synergy [131].

Biocompatible and biodegradable materials are also widely used in the preparation of microcapsules, among which polysaccharides and proteins are the main encapsulating materials [132]. For instance, some researchers have used *Lactobacillus*-derived membrane vesicles as biocompatible carriers to encapsulate the hydrophobic active substance fucoxanthin, and have prepared oral delivery vesicles with uniform particle size and strong stability. The delivery of fucoxanthin to the liver was achieved through intestinal epithelium-targeted adhesion and transcellular absorption mechanisms. In a high-fat diet-induced obesity mouse model, oral delivery vesicles achieve bidirectional regulation of lipid metabolism homeostasis in the liver by activating the AMPK signaling pathway in the liver. On the one hand, it down-regulates the expression of sterol regulatory element-binding protein 1c and its downstream fatty acid synthase and acetyl-CoA carboxylase 1, thereby inhibiting de novo lipid synthesis in the liver from the source. On the other hand, it up-regulates the expression of peroxisome proliferator-activated receptor α and carnitine palmitoyltransferase 1 A, enhancing the β-oxidation capacity of fatty acids in liver cell mitochondria and accelerating the catabolism of excessive lipids in the liver, directly reversing lipid deposition in liver cells [133]. The performance of microcapsules not only depends on the wall material and preparation process, but also the internal microstructure plays a decisive role in the encapsulation efficiency and release behavior. Microcapsules with dense structure and no holes can provide the best protection for probiotics, while microcapsules with loose structure or cracks are difficult to effectively maintain the activity of the strains. In addition, the material and size of microcapsules can hinder their penetration through the intestinal mucus layer, reducing the therapeutic effect on liver diseases.

#### Nano-delivery system

The emergence of nano-delivery systems represents a paradigm shift in the field of live bacteria therapy, transforming their role from mere protectors to precise-targeting intervention methods [134]. By combining engineered live bacteria with functional nano-materials, researchers have developed a new biological hybrid system. These structures not only enhance the survival ability and bioavailability of the bacteria in the gastrointestinal tract, but also give them the ability to precisely locate at specific diseased sites. Leveraging strong penetrating ability to traverse multiple physiological barriers. By layer-by-layer assembly of biocompatible polymer nanoshells onto bacterial surfaces, this strategy confers robust protection to viable bacteria against gastrointestinal stressors, including gastric acidity, proteolytic enzymes, and rapid luminal transit [135]. This coating strategy improves the survival rate of the bacteria and promotes intestinal attachment during oral administration. Moreover, the nano-delivery system can also design engineered live bacteria as drug carriers that can target tumor sites, thereby enhancing the therapeutic effect. These bacteria can selectively proliferate in the tumor microenvironment without causing infections or generating long-term adverse reactions, thus serving as a programmable treatment delivery platform. It is worth noting that some recent forward-looking studies have proposed strategies for using nano-armored bacteria for intestinal colonization. By taking advantage of the specific interaction between chitosan oligosaccharides and the inflammation-related protein CHI3L1, the adhesion and colonization of probiotics are promoted in the pathological microenvironment, solving the problem of low colonization efficiency of traditional probiotics [136].

Some studies have encapsulated engineered bacteria within nanoparticles, and through the targeting of nanoparticles, engineered bacteria can specifically accumulate in the tumor site. In particular, engineered living bacteria modified by genetic engineering or chemical materials can deliver PD-L1 and CTLA-4 in tumors to promote the treatment of diseases [137]. In addition, some researchers have used immunotherapy and synthetic biology to design living bacteria, combining immunotherapy expression with optimized lysis mechanism, so that living bacteria can carry nanobodies back to the core of necrotic tumors, grow to a critical density, and lyse, effectively and continuously releasing therapeutic drugs in the tumor microenvironment. For example, polydopamine-coated EcN carrying CRISPR/Cas9 plasmid-loaded liposome was used for enhanced immunotherapy of deep tumors [138]. At the same time, in the treatment of NAFLD and non-alcoholic steatohepatitis [139, 140], nanoparticles encapsulated living bacteria can affect liver lipid metabolism and inflammatory response by improving intestinal flora imbalance and regulating the production of intestinal metabolites, such as SCFAs. This delivery strategy not only improves efficacy, but also reduces the potential systemic side effects. Although nanoparticle encapsulation has many advantages, there are also many problems. For example, the preparation process of nanoparticles in large-scale production is complex, cost-prohibitive, and the encapsulation efficiency is difficult to control stably. In the future, the technology needs to be improved and upgraded to enable it to grow in clinical applications.

#### Single-cell encapsulation

Single-cell encapsulation technology realizes precise protection and functional modification of cells by constructing a nano-scale protective coating on the surface of a single living bacteria cell [141]. Different from the “collective protection” mode in which a large number of bacteria are encapsulated in the same matrix by traditional microcapsule technology, single cells are encapsulated into nanomaterials for each cell, thus maintaining the individual physiological activity of probiotics to the greatest extent and overcoming the problem of strain viability decline caused by mechanical or thermal damage during the preparation process in traditional methods [142]. Such nano-coatings are usually constructed from biocompatible polymers, biomimetic membranes, or layer-by-layer self-assembly techniques. The thickness is only nanoscale, which does not significantly increase the size of the bacteria or affect its application in food matrices. Single cell encapsulation technology gives living bacteria a series of superior performance. First, this layer of nanomaterials can bolster probiotic resilience to the hostile environment of the gastrointestinal tract, which contains gastric acidity, bile salts, and digestive enzymes, ensuring that a higher proportion of viable bacteria can reach the colon smoothly. Second, by modifying specific functional molecules on the surface of the coating, the adhesion and colonization ability can increase the survivability of probiotic strains, thereby supporting their function in the gut, such as gastric acid, bile salts, and the viability of probiotics on the intestinal mucosa can be enhanced, and their residence time in the intestinal tract can be prolonged, thus exerting a more lasting probiotic effect [142–144].

In the study of HCC treatment, a team tried to encapsulate the engineered bacteria with tumor suppressor function into single cells [145]. Through microfluidic technology, nanoparticles modified with tumor-targeting ligands on the surface are combined with a single engineered bacterial cell and encapsulated. Using the targeting of nanoparticles, the encapsulated engineered bacteria can specifically aggregate at the tumor site of HCC [145]. The surface functionalization of single cell encapsulated engineered bacteria can be used to reduce the immunogenicity of probiotics or achieve targeted recognition of specific lesions. For example, in the treatment of CRC and IBD, single-cell encapsulated living bacteria may have certain application prospects. The living bacteria encapsulated by single cells can also enhance the intestinal colonization ability and function of living bacteria. The single-cell encapsulation of *Bifidobacterium animalis* subsp. *lactis* via inulin and metal phenolic networks improved its tolerance to gastric acid and bile salts and antioxidant capacity, and prolonged intestinal retention time by hydrogen bonding with intestinal mucosa [146, 147]. However, single-cell encapsulation still requires addressing issues related to quality control and coating stability. Due to its technical instability and high production costs, its clinical application remains limited.

In conclusion, we have summarized the advantages and limitations of the structure of engineered bacteria and their delivery methods to facilitate their better application in disease treatment (Table 2).

**Table 2:** Advantages and disadvantages of the structural design of live bacteria

Delivery Method	Core Structure	Advantages	Disadvantages	Application method	Reference
Capsules	-Live bacteria encapsulated in shells, which slowly disintegrate and release in the gastrointestinal environment to avoid direct damage by gastric acid	-Simple preparation -Processeasy administration -Low cost	-Weak protective ability, no targeting -Difficult to precisely control release rate	-Oral administration	[125]
Microencapsules	-Live bacteria encapsulated in micrometer-scale semipermeable membrane vesicles, the membrane prevents external environmental interference and enables on-demand responsive release	-Excellent protective performance -High bacteria loading capacity -Controllable release	-Vesicles tend to agglomerate -Some materials are prone to degradation by intestinal flora	-Oral administration	[148]
Nano-delivery system	-Live bacteria encapsulated/adsorbed by nanoscale carriers, which accumulate at targets via the Enhanced Permeability and Retention effect or ligand-mediated targeting	-Strong targeting ability -Good permeability -Excellent immune evasion capability	-Complex preparation process -Low bacteria loading capacity	-Injection administration -Oral administration	[149, 150]
Single-cell encapsulation	-Individual live bacterial cells wrapped in ultra-thin nanoscale membranes, forming independent protective units to avoid interactions between bacteria and external interference	-Extremely high protection precision -Precisely controllable release rate	-Exceptionally high preparation cost -High difficulty in mass production -Membrane structure prone to damage by mechanical forces	-Oral administration -In vitro cell culture	[141, 151]

## Application in liver diseases

Traditional drugs for treating liver diseases mainly suffer from inherent limitations such as insufficient targeting, low bioavailability, and systemic toxicity [152]. Oral administration requires absorption through the gastrointestinal tract and first-pass metabolism in the liver, resulting in the actual concentration of the drug in the target tissue being much lower than the administered dose. Intravenous administration can bypass the absorption barrier, but it is difficult to achieve precise identification of liver parenchymal cells or specific non-parenchymal cells. Moreover, chronic liver diseases require long-term and repeated administration, which is prone to induce drug resistance and accumulation toxicity, further limiting the clinical application of traditional drugs. Based on the breakthroughs in the gene modification, covalent connection, and structural design levels of the live bacterial engineering strategy mentioned above, engineered live bacteria offer a strategy. First, as a core metabolic organ of the human body [153], the liver is rich in the reticuloendothelial system and the portal venous circulation system. This enables engineered bacteria administered orally or via intravenous injection to naturally accumulate in the hepatic sinusoids. This passive process, rather than active targeting, benefits from the liver’s unique anatomical structure; second, the liver, as an intrinsic immune tolerance, has a natural immune tolerance to commensal microbes and their components, preventing the engineered bacteria from being excessively cleared by the immune system in the liver; third, through gene editing and surface modification technologies, the engineered bacteria can actively respond to the pathological microenvironment, maximizing efficacy and reducing the risk of systemic exposure. This chapter will systematically elaborate on the application progress of engineered live bacteria in HCC, cirrhosis, NAFLD and ALD, viral hepatitis, and diseases related to the gut-liver axis.

### Hepatocellular carcinoma (HCC)

From the perspective of global incidence rates, HCC is currently one of the main causes of death related to malignant tumors worldwide. Although surgical procedures and local ablation have made progress in recent years, traditional chemotherapy drugs and molecular targeted drugs, such as sorafenib, are limited in clinical application [154]. This is often due to the insufficient concentration of drugs that accumulate in the tumor site after absorption, as well as the severe adverse reactions caused by their non-selective killing effect on normal tissues, resulting in poor patient tolerance. In this context, tumor treatment strategies based on engineered bacteria offer a reference for exploring solutions to the limitations of existing therapies for HCC. Firstly, the modified bacteria have the inherent ability to target hypoxic areas of tumors and can achieve high-density colonization within the tumors. They can continuously release therapeutic molecules in the body, thus solving the obstacles of traditional drug delivery methods and the insufficient exposure of drugs to the lesion sites [155]. For example, VNP20009 with an ultrasound-responsive gene circuit carrying an IFN-γ therapeutic payload is administered via intravenous injection and actively moves towards liver tumor lesions. Triggered by ultrasound, IFN-γ is released in the body, thereby achieving a high complete tumor remission rate in HCC models [156]. Moreover, engineered bacteria can reshape the immune microenvironment of HCC and reverse immunosuppression. Studies have shown that specific intratumoral bacteria, such as *B. subtilis*, can regulate lipid metabolism, promote the acetylation of retinoic acid receptor-related orphan receptor gamma in NK cells, thereby inducing the ubiquitin-mediated degradation of iron transporters and inhibiting NK cell ferroptosis, thereby enhancing their anti-tumor immune response [157].

**Fig. 8:**
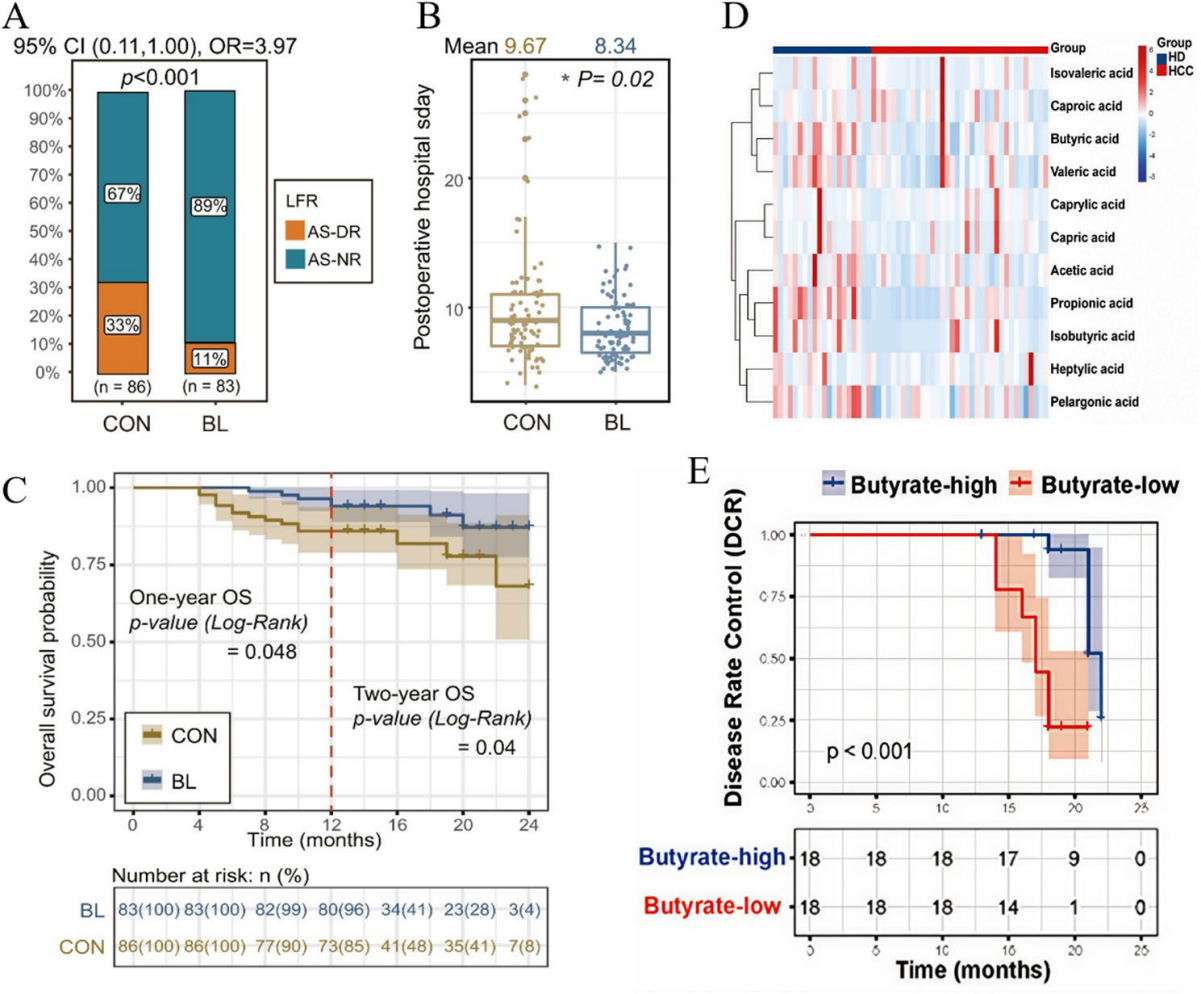
(**A**) The LFR rate was compared between the control (CON) and *Bifidobacterium longum* (BL) groups using the chi-square test. (**B**) Analysis of postoperative hospitalization length (assessed by chi-square test) in the CON versus BL groups. (**C**) Kaplan-Meier estimate of the overall survival (OS) probability in HCC patients. Copyright^©^2024 Cell Host Microbe. (**D**) Heatmap illustrating alterations in human plasma SCFAs in healthy donors and HCC patients. (**E**) Progression-free survival was compared between patients with high and low butyrate levels. Copyright^©^2025, Gut Microbes

*Bifidobacterium* has also shown therapeutic potential as adjuvants in the adjuvant treatment of HCC. Oral administration of this strain during the perioperative period can upregulate proliferation markers Cyclin D1 and Ki67, promote postoperative liver function recovery [158], reduce postoperative complications, lower the risk of delayed recovery, and shorten hospital stays (Fig. 8A-C). Moreover, a research team constructed a shuttle expression vector named pBLES100-S-eCD for EcN-*Bifidobacterium*. Following electroporation-mediated transfer into *Bifidobacterium*, the resulting engineered strain specifically colonized tumors in a rat xenograft model and expressed the cytosine deaminase enzyme, which converted the 5-fluorocytosine into the cytotoxic agent 5-fluorouracil, inducing tumor cell apoptosis and regression. Besides *Bifidobacterium*, *L. reuteri* can also exert anti-tumor effects by regulating tryptophan metabolism, reducing the tumor incidence in mice with intestinal flora imbalance [159] (Fig. 8D-E). Furthermore, in a murine HCC model, engineered bacteria EcN were used as targeted delivery vectors. Researchers delivered anti-angiogenic protein Tum-5 and tumor suppressor protein p53 to the hypoxic regions of tumors through engineered bacteria EcN, thereby inhibiting tumor progression [160]. Collectively, these preclinical studies underscore the promise of engineered live bacterial therapeutics for HCC treatment and provide references for translational development.

### Cirrhosis

**Fig. 9:**
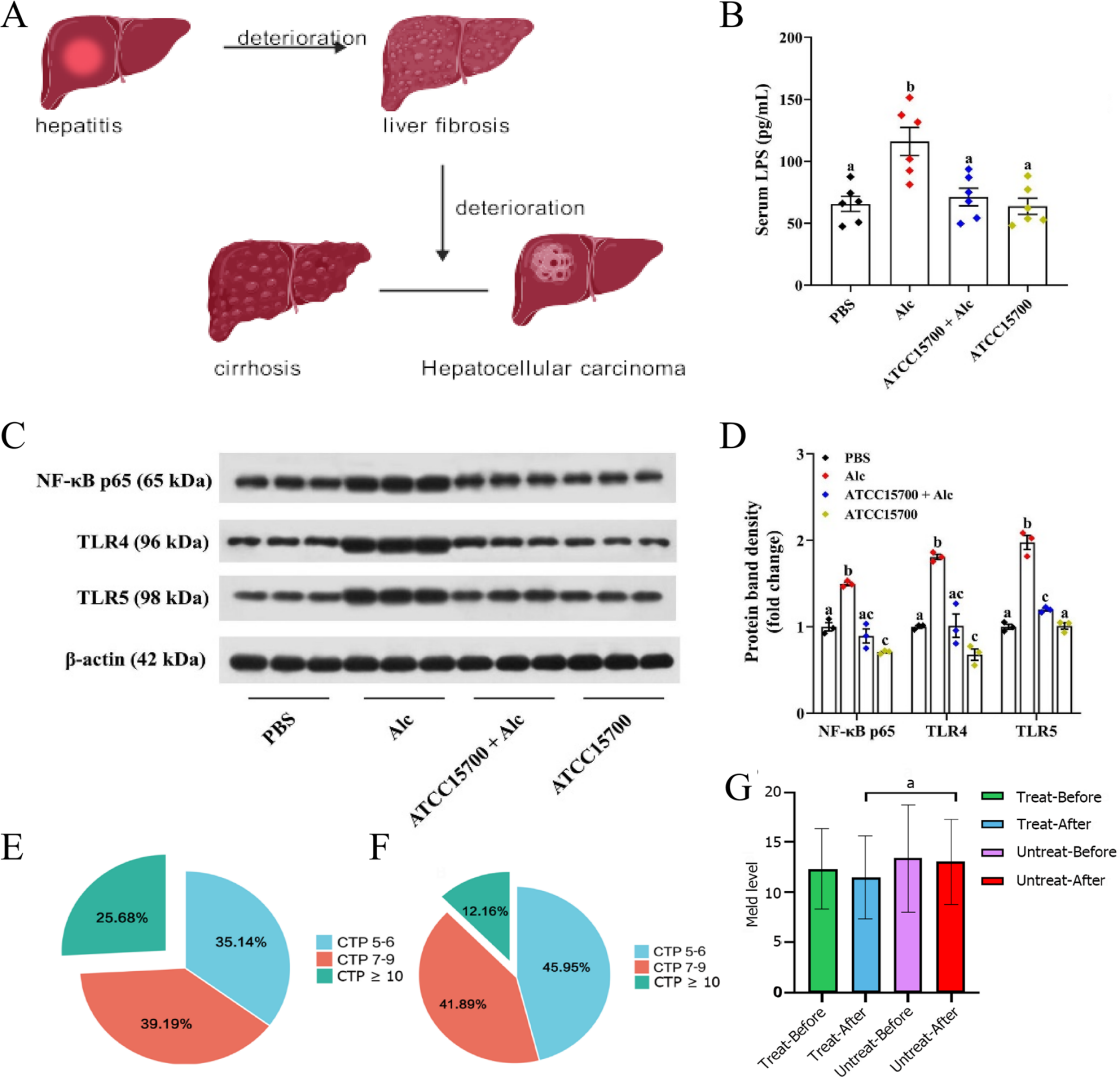
(**A**) The process of cirrhosis (created by BioRender.com). (**B**) Serum LPS levels. (**C**) Western blotting of hepatic NF-κB, TLR4, and TLR5. (**D**) Liver protein quantification study. Copyright^©^2020, Journal of Functional Foods. (**E**) The baseline distribution of intervention group across Child-Turcotte-Pugh grades A, B, and C was assessed prior to treatment. (**F**) Following administration of *Lactobacillus paracasei* N1115, the distribution of Child-Turcotte-Pugh grades A, B, and C among patients in the treatment group was assessed. (**G**) MELD scores were compared across both cohorts. Copyright^©^2024, World J Gastroenterol

Cirrhosis is the irreversible, advanced stage of chronic liver disease, characterized by chronic inflammation, hepatocyte degeneration, and progressive fibrosis [161]. It is often accompanied by multiple complications and deteriorating conditions (Fig. 9A). Currently, the treatment strategies for cirrhosis mainly focus on controlling the underlying causes, such as antiviral therapy and alcohol cessation. However, these interventions are mostly symptomatic treatments that are difficult to effectively reverse the process of liver fibrosis and cannot repair the already compromised liver structure and function. Therefore, cirrhosis patients urgently need new treatment strategies. Researchers have discovered that engineered bacteria can target the gut-liver axis and improve metabolic disorders. Cirrhosis portal hypertension often leads to intestinal barrier dysfunction and microbiota imbalance. Microbiota-derived toxins enter the liver through the portal vein and are key drivers of disease progression and the occurrence of complications [162]. Orally administered engineered bacteria colonize the intestinal lumen, enabling localized intervention. For example, engineered EcN is designed to efficiently metabolize intestinal ammonia and convert it into beneficial amino acids such as L-arginine, thereby systematically reducing blood ammonia levels [163].

Live bacteria therapy, especially engineered bacteria treatment, offers a choice for the treatment of cirrhosis. For instance, *Bifidobacterium breve* ATCC 15,700 alleviates the continuous damage caused by intestinal bacterial translocation by enhancing intestinal barrier function, inhibiting the secretion of inflammatory mediators and regulating immune homeostasis [164], thereby delaying disease progression (Fig. 9B-D). Additionally, clinical studies have shown that the treatment of cirrhosis with live bacteria intervention increases the alpha diversity of the intestinal microbiota in patients [165], restores the dominant colonization of beneficial bacteria that produce SCFAs, and inhibits the overgrowth of pathogenic bacteria such as *Enterobacteria*. The favorable results of liver function indicators and the model for end-stage liver disease scores in patients also support this effect (Fig. 9E-G). In terms of engineered bacteria modification, previous studies have successfully constructed a genetically engineered bacterium EcN-MT that can stably express metallothionein by electroporation of the recombinant pET-28a-MT plasmid into the competent cells of EcN. Chronic inflammation can lead to an increase in the expression of α-smooth muscle actin, activate hepatic stellate cells, and cause collagen deposition in liver tissue, thereby inducing fibrosis. Liver fibrosis is an intermediate stage in the further development of cirrhosis. Supplementation with EcN-MT can reduce liver inflammation and the expression of α-smooth muscle actin, and impede fibrosis progression [166].

### Non-alcoholic fatty liver disease (NAFLD) and alcoholic liver disease (ALD)

NAFLD is one of the most prevalent chronic liver conditions worldwide, affecting more than a quarter of adults globally. NAFLD often coexists with metabolic syndrome, and traditional drugs have difficulties in balancing liver protection and metabolic regulation, with varying treatment effects due to individual differences. Given the complex pathogenesis of NAFLD, which involves insulin resistance, abnormal nuclear receptor signaling pathways, genetic risk factors, and changes in the gut microbiota, current treatment options remain limited. The current traditional treatment methods mainly focus on lifestyle intervention, supplemented by insulin sensitizers and vitamin E, but the treatment effects are often limited. It is worth noting that engineered bacteria can be designed to target key nodes of lipid synthesis and breakdown in NAFLD [167, 168]. Preclinical studies demonstrate that specific engineered bacteria or functional strains can work together: on the one hand, they can enrich beneficial bacteria such as butyrate-producing bacteria; on the other hand, they can activate lipid oxidation pathways such as AMP-activated protein kinase and carnitine palmitoyltransferase 1α in the liver, and inhibit lipid synthesis key factors such as fatty acid synthase and sterol regulatory element-binding protein 2, thereby reducing systemic lipid levels in the host, decreasing adiposity, and ameliorating hepatic steatosis [169].

**Fig. 10:**
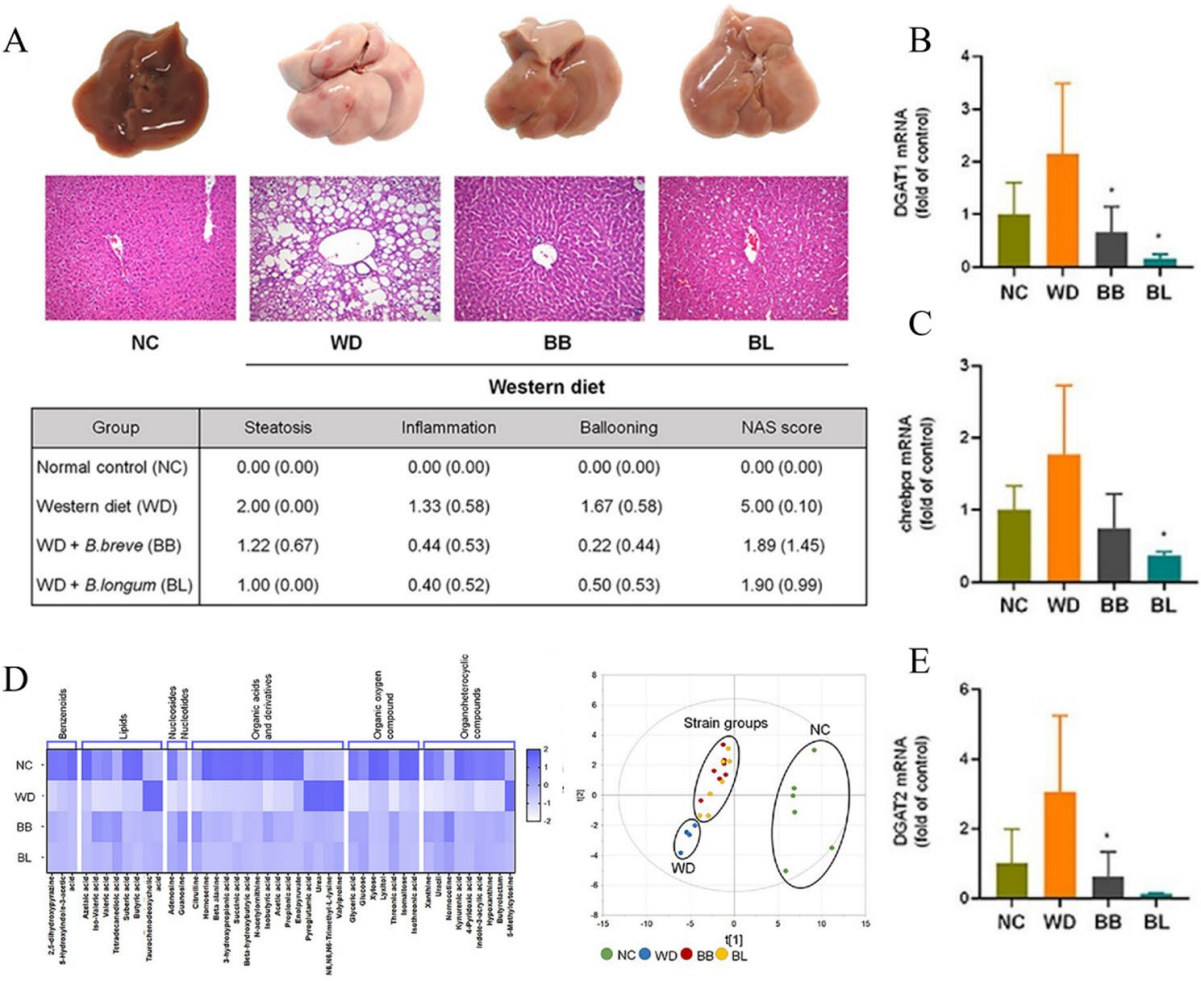
(**A**) Panels show representative liver samples from the *Bifidobacterium* group and three control groups: the top displays gross morphological findings, while the bottom presents histological sections stained with hematoxylin and eosin. (**B**) mRNA levels of DGAT1. (**C**) mRNA levels of chrebpα. (**D**) Illustrating the overlapping metabolites, a heat map assigns them to seven super classes, and a principal component analysis scatter plot reveals their core compositional relationships. (**E**) mRNA levels of DGAT2. Copyright^©^2019, J Hepatol

*Bifidobacterium* has played a significant role in the treatment of NAFLD. Studies have shown that after long-term oral administration of the bacteria, liver fat content and serum triglyceride levels decreased, and insulin resistance index also improved a lot (Fig. 10A-C). *B. subtilis* also holds value in the treatment of NAFLD due to its well-characterized genetic background. Wild-type *B. subtili*s primarily produces butyrate via the butyrate kinase pathway, which is inefficient, and its colonization and metabolic activity within the host gut are limited by its growth rate. To address this, researchers engineered *B. subtilis* SCK6 by knocking out the *skfA* gene and inserting the *BCoAT* gene, thereby introducing a highly efficient novel pathway for butyrate synthesis [170]. The engineered bacterial formulation was administered daily via oral gavage into the intestines of a high-fat diet-induced obese mouse model. This engineered strain effectively reduced blood lipid levels, improved glycemic control and insulin resistance, and subsequently alleviated hepatic steatosis in the model mice. Furthermore, it ameliorated multiple metabolic pathways associated with NAFLD. The improvement in NAFLD is primarily attributed to two mechanisms. First, it regulates bile acid metabolism, as butyrate upregulates expression of hepatic cholesterol 7α-hydroxylase, a key rate-limiting enzyme in bile acid synthesis, thereby improving lipid metabolism and glucose homeostasis. Second, concerning oxidative stress and inflammation, butyrate enhances the activity of antioxidant defense systems such as superoxide dismutase, alleviating high-fat diet-induced hepatic oxidative damage and the expression of inflammatory factors [171], 172].

Strategies targeting gut microbes, such as regulating metabolites such as intestinal microbial enzymes, bile acids, and SCFAs, will promote the development of personalized treatment for NAFLD. Elucidating the mechanisms underlying the host-gut microbiome epigenetic dialogue will facilitate novel strategies to prevent and treat NAFLD and its associated comorbidities. For ALD, in vivo bacterial therapeutics have emerged as an adjunct to standard care to ALD treatment by reshaping intestinal microecology and blocking the vicious cycle. *Lactobacillus plantarum* demonstrates efficacy in the early intervention of ALD [173]. Beyond the mechanisms previously discussed, *Bifidobacterium* may have therapeutic effects on NAFLD and ALD through its regulatory role on gut metabolites [174]. These bacteria help restore metabolic homeostasis, with this regulatory capacity spanning diverse metabolite classes. (Fig. 10D-E). The key to research on liver diseases such as ALD lies in elucidating the mechanisms governing the gut microbiome-immune system interplay predominant. Such mechanistic insights are helpful in providing a theoretical basis for the design of engineered live bacteria.

### Viral hepatitis

Viral hepatitis constitutes a significant public health challenge worldwide. HBV serves as a representative example: upon hepatocyte invasion, it establishes a persistent infection by forming covalently closed circular DNA in the nucleus, a stable transcriptional template that drives viral gene expression and replication. The virus induces functional exhaustion of specific CD8 + T cells through high antigen load and upregulates inhibitory receptors such as PD-1, thereby preventing the immune system from effectively clearing the virus [175, 176]. However, drug treatments for viral hepatitis cannot eliminate closed circular DNA, and have limited effect on increasing the rate of hepatitis B surface antigen seroconversion [177], 178]. Fortunately, genetically engineered live bacteria have improved this situation. Through synthetic biology plasmid construction and surface display system engineering strategies, the live bacteria are modified to stably express anti-viral neutralizing nanobodies or viral antigens on their surface. The outer membrane vesicles naturally secreted by Gram-negative bacteria can be used to achieve systemic delivery of therapeutic molecules across the intestinal barrier, with both passive antiviral treatment and active immune protection functions. These modified bacteria can directly capture and neutralize virus particles. Moreover, these genetically modified bacteria can continuously deliver immune regulatory molecules to the gut [179].

Studies have shown that *Bifidobacterium animalis* BB-12 has beneficial effects in the treatment of HBV infection. Specifically, the combination of entecavir and *Bifidobacterium animalis* BB-12 in the treatment of patients with HBV leads to a decrease in hepatitis B surface antigen titer, an increase in the seroconversion rate of HBeAg, and no other adverse reactions [180]. In addition, *Akkermansia* has potential value in the treatment of hepatitis C. The abundance of *Akkermansia* in the intestines of patients with hepatitis C is reduced, and it is negatively correlated with the degree of liver fibrosis. Supplementing *Akkermansia* can alleviate liver fibrosis, and the potential mechanism may include increasing the thickness of the intestinal mucus layer, reducing bacterial translocation, and regulating bile acid metabolism. Collectively, these findings in viral hepatitis provide mechanistic rationale and translational foundations for developing engineered live bacterial therapeutics.

### Diseases related to the gut-liver axis

Gut microbiota can affect the liver and the human body through the gut-liver axis. Diseases related to the gut-liver axis refer to liver diseases mediated by the dysfunction of the gut-liver axis. The core types include typical chronic cholestatic diseases, especially primary biliary cholangitis, primary sclerosing cholangitis, and acute-on-chronic liver failure [4, 181]. Current pharmacotherapies for these disorders remain suboptimal. For instance, the standard treatment regimens, such as immunosuppressants and ursodeoxycholic acid (UDCA), may not be able to prevent the continuous progression of the patient’s condition. Moreover, prolonged immunosuppressant use predisposes patients to metabolic disturbances and heightened infection risk.

In summary, all the above content indicates that a new therapeutic approach is needed to deal with diseases related to the gut-liver axis. Currently, studies have engineered EcN by optimizing the bile acid 7α-dehydratase baiE gene from *Clostridium scindens* and constructed a recombinant expression vector that can stably express the BaiE protein. This vector was then transferred into EcN to obtain the engineered strain EcN-BaiE. This engineered live bacteria can activate the intestinal FXR-fibroblast growth factor 15/19 signaling pathway, alleviate vancomycin-induced bile acid accumulation and liver fibrosis. It is expected to become a treatment method for patients with primary sclerosing cholangitis [182]. In addition, other live bacteria have also shown effective roles in the clinical treatment of diseases related to the gut-liver axis. For example, *Lactobacillus* and *Bifidobacterium* preparations can inhibit the TLR2/4 signaling pathway, down-regulate IL-33 expression, and correct IL-33-mediated Treg/Th17 immune imbalance. At the same time, they can restore the stability of the intestinal microbiota, reduce liver inflammation and injury through the gut-liver axis, and ultimately alleviate the progression of autoimmune hepatitis. These effects have been verified in animal models of AIH [183]. In patients with primary biliary cholangitis, specific strains such as *Lactobacillus plantarum* can improve the therapeutic response to UDCA and relieve pruritus. *S. boulardii* can enhance the tolerance of treatment in patients with acute-on-chronic liver failure. These live bacteria provide reference for research on diseases related to the gut-liver axis. The relevant live bacteria can be engineered to enhance their therapeutic effects on diseases related to the gut-liver axis.

### Comparison of bacterial vectors and delivery systems

Research on engineered live bacteria encompasses both expression platforms and delivery systems, which collectively determine their therapeutic capacity and the feasibility of clinical translation. Among the current mainstream expression platforms, EcN is widely favored for modification due to its well-defined genetic background and mature manipulation tools. However, the inherent risks associated with Gram-negative bacteria, namely lipopolysaccharide endotoxicity and the potential for plasmid-mediated horizontal gene transfer, remain obstacles to its application in patients with cirrhosis or immunocompromised conditions. *B. subtilis*, a spore-forming Gram-positive bacterium, possesses excellent acid and heat tolerance along with efficient protein secretion capabilities. Nevertheless, its utility as a delivery vector for precision therapy is affected by difficulties in engineering, particularly concerning eukaryotic expression systems. In contrast, human gut commensal probiotics, represented by *Bifidobacterium* and *L. reuteri*, constitute the safest carriers for intervening in chronic liver diseases associated with the gut-liver axis. They pose no endotoxin risk, have a well-documented history of safe consumption, and achieve multi-targeted chronic disease intervention by regulating the intestinal microecology, repairing the gut barrier, and reducing the influx of hepatotoxic metabolites into the liver [184]. *S. boulardii* possesses eukaryotic post-translational modification capabilities and natural antibiotic resistance. However, its intestinal retention time is short, and its bile salt tolerance is relatively weak. Furthermore, there is a risk of fungemia in immunocompromised patients [185].

**Fig. 11:**
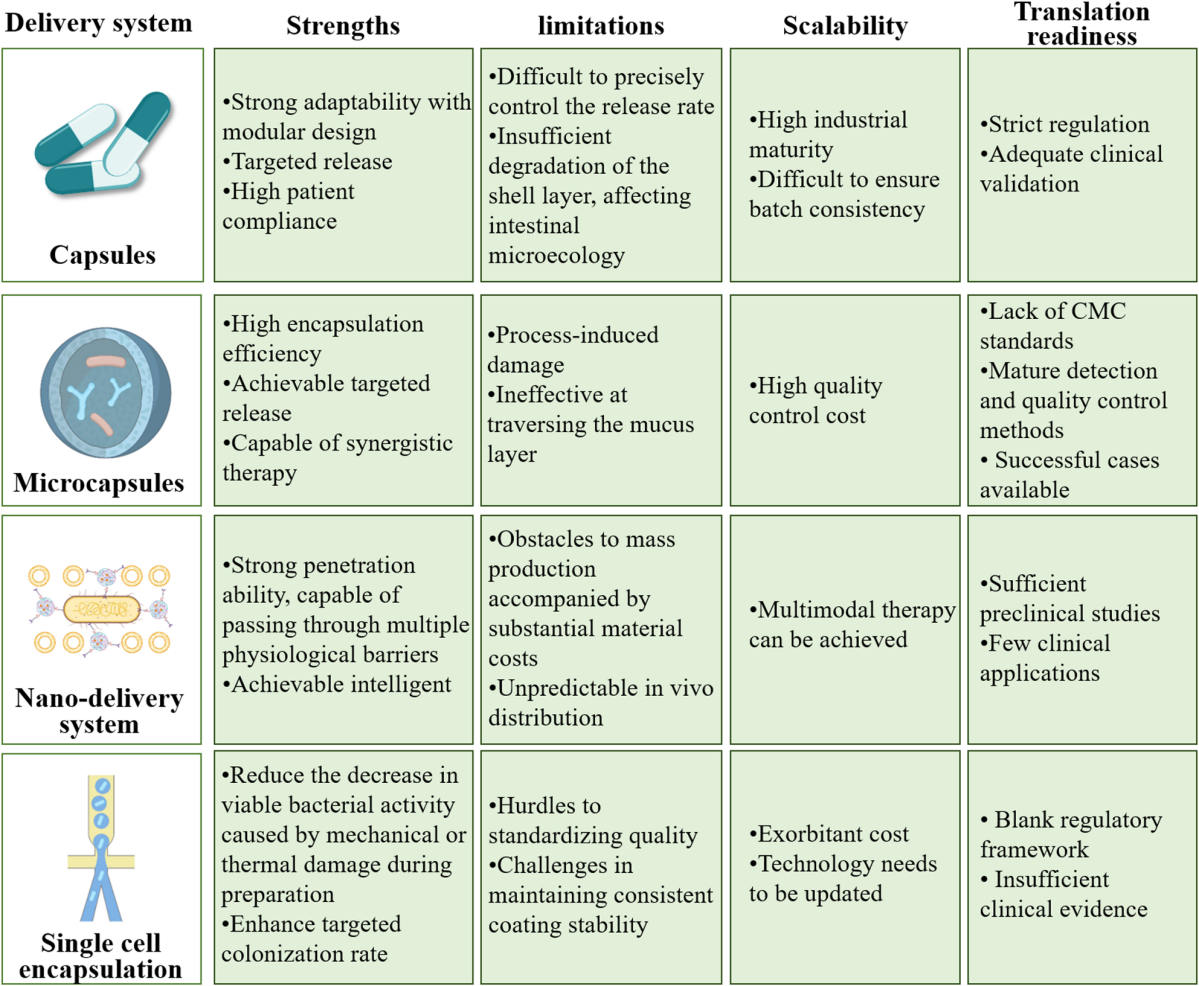
Engineered live bacteria and their delivery systems (created by BioRender.com)

The delivery method of engineered live bacteria products plays a critical role in their therapeutic efficacy, scalability, and translation readiness (Fig. 11). Capsules and microcapsules are currently the most intensively researched technological routes. Capsule delivery stands as the most industrially scalable strategy for live bacterial delivery. It supports modular design and targeted intestinal release, offers excellent patient compliance, and benefits from a mature regulatory framework and substantial clinical validation. However, achieving precise control over release kinetics with capsules is challenging, and incomplete degradation of the shell can potentially disrupt intestinal homeostasis [127, 186]. Furthermore, this system offers only physical isolation and cannot protect the bacteria from degradation by bile or digestive enzymes, nor from colonization competition posed by the native gut microbiota. Despite mature production technologies, ensuring batch-to-batch consistency remains difficult. Microcapsules offer high encapsulation efficiency, enable site-specific release, and can be utilized for synergistic therapies. However, the preparation process often compromises bacterial viability, and the relatively large particle size impedes effective penetration of the mucus layer. Their release behavior is highly dependent on the gut microbiota, resulting in poor controllability and high costs for quality control [187]. While unified chemistry, manufacturing, and controls standards are yet to be established, detection and quality assessment methods are relatively mature and are supported by successful preclinical cases.

Nanodelivery systems exhibit strong penetrative capabilities, enabling microenvironment-responsive release and multimodal combination therapies. Their primary drawbacks include high material costs, difficulties in large-scale production, and unpredictable in vivo biodistribution. The potential toxicity of nanomaterials also requires careful consideration. Despite extensive preclinical research, very few formulations have progressed to clinical trials [188]. Single-cell encapsulation is an emerging strategy that minimizes mechanical or thermal damage to live bacteria during preparation, enhances targeted colonization capacity, and reduces the risk of HGT through physical isolation of engineered strains. However, this approach formidable quality control hurdles, challenges in maintaining coating stability, and high production costs, relying on novel manufacturing technologies [189]. Currently, it lacks a defined regulatory framework and is entirely devoid of clinical data, remaining confined to fundamental research.

## Challenges and perspectives

### Clinical translation

In recent years, engineered bacteria have emerged as a promising platform technology for treating liver diseases due to their ability to passively distribute in liver tissues, modulate immune responses, and alleviate metabolic abnormalities. It is critical to distinguish between preclinical findings and clinical evidence when evaluating engineered live bacteria therapies. Preclinical studies, primarily conducted in rodent models, provide essential proof-of-concept data regarding mechanism of action, biodistribution, and preliminary efficacy. However, these models have inherent limitations, including differences in gut microbiota composition, immune system architecture, and metabolic pathways between rodents and humans. Furthermore, the standardization of preclinical models varies considerably across studies, complicating cross-study comparisons and reproducibility. Clinical evidence for engineered live bacteria in liver diseases remains in early stages. While several phase I/II trials have demonstrated safety (Table 3), efficacy data from large-scale, randomized, placebo-controlled trials are largely unavailable. The translation gap between promising preclinical results and clinical validation is substantial, as evidenced by the limited number of engineered bacterial products that have progressed beyond early-phase trials. Future research must prioritize rigorous clinical validation while acknowledging that preclinical findings, however promising, do not guarantee clinical success.

**Table 3:** The preclinical and clinical trials of engineered live bacteria

	Live bacteria	Engineered bacteria	Disease	Application method	Clinical trial number	Reference
**Clinical trials**	*Listeria monocytogenes*	Knock out the msbB and purI genes and introduce the human interleukin-2 expression cassette	Liver metastatic tumor	Intravenous injection	NCT00327652	[190]
	*Salmonella*	Dual attenuation achieved through deletion of the internalin B and actin-assembly factor A	HCC	Oral administration	NCT01099631	[191]
	EcN	Knock out the thyA gene and introduce the ammonia metabolism enzyme system	Cirrhosis	Oral administration	NCT03447730	[163]
	*Bifidobacterium*	Transfer the plasmid DNA carrying the human IL-12 expression cassette	Solid tumors	Oral administration	NCT04025307	[192]
	EcN	Insert the dacA gene derived from *Listeria monocytogenes*	Advanced solid tumors	Intratumoral injection	NCT04167137	[193]
**Preclinical** **trials**	*B. subtilis* SCK6	Knock out the skfA gene and insert the BCoAT gene	NAFLD	Oral administration	**/**	[170]
	EcN	Transfer into the recombinant expression vector that can stably express the BaiE protein	Diseases related to the gut-liver axis	Oral administration	**/**	[182]
	*L. reuter*	Modification of lipid nanoparticles onto the bacterial surface was achieved via a ROS-cleavable linker	Liver metastases of CRC	Oral administration	**/**	[194]
	EcN	Loading ICG onto zinc-based metal-organic frameworks enables the modification of EcN-Nb	Solid tumors	Tail vein injection	**/**	[105]
	*Bifidobacterium bifidum*	Covalently modify the surface of bacteria with doxorubicin-loaded calcium phosphate/silica nanoparticles	Solid tumors	Tail vein injection and intratumoral injection	**/**	[138]
	*L. reuteri* ATCC PTA 6475	Fusion of the IL-22 gene with a signal peptide was cloned into the high-copy plasmid pJP028 and transformed into *L. reuteri*	NAFLD	Oral administration	**/**	[195]
	EcN	Bacterial surface display technology for delivering metallothionein	Cadmium-induced liver injury	Oral administration	**/**	[196]

Several live bacterial therapies have entered clinical trials, indicating translational potential for this treatment approach. In the field of HCC treatment, engineered bacteria have achieved results. For instance, the VNP20009 strain [190], a genetically modified strain expressing IL-2, was tested in a phase I clinical trial (NCT01099631). Researchers achieved attenuation by knocking out the msbB and purI genes and introduced a human IL-2 expression cassette, aiming to achieve tumor-selective colonization and local immune activation while minimizing systemic toxicity. Furthermore, researchers have developed the CRS-100 strain [191], a vaccine therapy based on attenuated *Listeria monocytogenes*, for the treatment of liver metastases (NCT00327652). This strain was doubly attenuated, and some derivative strains (such as CRS-207) further expressed the tumor-associated antigen mesothelin. By intravenous injection, it utilized the natural ability to infect antigen-presenting cells in the liver, triggering adaptive immune response in the liver microenvironment.

In the field of cirrhosis research, the SYNB1020 strain [163], derived from EcN and capable of consuming ammonia, has been used in the treatment of cirrhosis and hyperammonemia (NCT03447730). This strain was made thymine auxotrophic by knocking out the thyA gene and introduced an ammonia metabolism enzyme system. It converts ammonia in the gut into arginine, thereby reducing blood ammonia levels. Besides the above liver-related applications, engineered bacteria targeting solid tumors are also closely related to liver tumor treatment. bacTRL-IL-12 is a platform technology based on *Bifidobacterium longum* [192], which constructs a recombinant strain expressing human IL-12 and utilizes the natural ability of *Bifidobacterium* to target hypoxic regions of tumors to directly deliver IL-12 to the tumor microenvironment. This drug has been evaluated in a phase I study for advanced solid tumors (NCT04025307). Similarly, SYNB1891 is an engineered EcN strain that expresses the DacA enzyme from *Listeria monocytogenes* to produce cyclic di-adenosine monophosphate under hypoxic conditions [193], thereby activating the stimulator of interferon genes pathway. Currently, this study has entered the clinical trial stage for advanced solid tumors and lymphomas (NCT04167137). Both of these drugs demonstrate the certain application potential of tumor-colonizing bacteria in the treatment of primary HCC and metastatic HCC.

In addition to the above-mentioned clinical studies, many engineered bacteria have demonstrated therapeutic effects in preclinical research. Both *L. reuteri* ATCC PTA 6475 and *B. subtilis* SCK6 have shown good therapeutic effects in the treatment of NAFLD. Among them, *L. reuteri* ATCC PTA 6475 has been successfully engineered into a recombinant strain capable of secreting IL-22 [195], which achieves in situ delivery of the cytokine in the intestinal tract through the high-copy plasmid pJP028, demonstrating superior lipid-lowering effects in NAFLD models compared to the wild-type strain. Additionally, *L. reuteri* has also been applied in the field of tumor treatment [194]. Researchers have constructed the LR-S-CD/CpG@LNPs system by modifying its surface with silkworm leaf lipid nanoparticles co-loaded with carbon dots and the immune adjuvant CpG through an active oxygen cleavable linker, which can effectively inhibit the progression of primary CRC and its liver metastases through photodynamic immunotherapy. In the fields of toxic liver injury, disease related to the gut-liver axis, and tumor treatment, the modification research of EcN is the most active. For instance, EcN-MT [196], constructed by anchoring metallothionein on the bacterial surface through surface display technology, not only reduces the liver cadmium content in a cadmium-exposed mouse model but also improves liver steatosis, inflammatory infiltration, and fibrosis symptoms by activating the Nrf2-Keap1 pathway. Moreover, other microbial carriers also exhibit unique advantages. For example, *Bifidobacterium bifidum* [138], with its hypoxia tropism, has been used to covalently modify calcium phosphate/silica nanoparticles loaded with doxorubicin, constructing the DNPs@Bi system that can actively target chemotherapy drugs to the hypoxic core of tumors, achieving effective killing of solid tumors through the combination of chemotherapy and immunogenic cell death effects.

These advancements indicate that engineered live bacteria hold value in the treatment of liver diseases, particularly in the field of precise targeted therapy. However, to bridge the gap from the laboratory to clinical application, it is necessary to pay attention to the differences between animal models and human data [197]. The complexity of biological barriers between species and the limitations of model systems in simulating the real physiological environment of humans can lead to differences in the therapeutic effects of engineered live bacteria in clinical applications. For instance, the transduction efficiency of human macrophages mediated by lentiviral vectors is extremely low, and this barrier directly hinders the transformation of gene therapy based on macrophage modification from rodents to clinical practice. To address this issue, researchers are working on developing translational research platforms that can more accurately simulate human physiological and pathological conditions [198]. By constructing humanized animal models to modify the host, such as transplanting human hematopoietic stem cells or human liver cells into immunodeficient mice to carry functional human immune cells or target organs, the interaction between engineered bacteria and human cells can be evaluated in a living environment. In addition to this issue, the clinical translation of engineered live bacteria also needs to address key problems such as the genetic stability of strains, long-term assessment of biological safety, and risk control for immunodeficient populations, and establish a complete regulatory system.

### Biosafety and horizontal gene transfer

The application of engineered live bacteria in liver diseases, from laboratory research to clinical practice, requires the establishment of a corresponding safety guarantee system to balance efficacy and risk, and ultimately achieve safe and effective transformation and application. Although genetic engineering can endow bacterial strains with specific therapeutic functions, engineered live bacteria still face certain biosafety issues when entering the host or environmental system. Firstly, engineered bacterial strains carrying designed genetic circuits may undergo mutations due to host environmental stress, leading to gene loss or failure of safety designs [199]. The kill switch of engineered live bacteria is difficult to maintain stability due to the application of strong selective pressure, and there may be a risk of escape mutations in the bacterial community in complex environments. Some studies have constructed CRISPR-based microbial kill switches [200], with an escape frequency of less than 10^− 8^ under laboratory pure culture conditions. However, in the complex environment of the mouse intestine, a large number of escape strains were detected within just 7 days. Secondly, engineered bacteria may express uncontrollably and over-activate the host immune system, leading to inflammatory responses, sepsis or autoimmune reactions [201]. In addition, engineered bacteria may survive and reproduce in non-target environments, causing environmental impacts. After oral or intravenous administration, engineered live bacteria may colonize and proliferate in non-target sites, making it impossible to achieve spatiotemporal control for their clearance [202]. For example, VNP20009, which has been genetically modified for tumor treatment, was detected in the liver, spleen, and bone marrow of animal models after use [203]. All these factors make the biosafety of engineered live bacteria difficult to control.

To address the biosafety issues of the aforementioned engineered bacteria, researchers have proposed a variety of design methods. For instance, site-specific integration technology is adopted to insert the target gene into a specific site of the bacterial genome, while eliminating potential toxic genes or competing related genes to ensure that the bacteria do not undergo unexpected mutations or gene deletions [204]. Alternatively, an inducible control system can be designed, such as using arabinose [205], tetracycline, etc., to achieve a promoter-based switch for precise spatiotemporal regulation of gene expression. Additionally, conditional lethal switches or auxotrophic markers can be introduced to design the engineered bacteria to survive only in the presence of specific synthetic nutrients [206].

One of the challenges in the clinical translation of engineered live bacteria is the risk of horizontal gene transfer. The therapeutic genes or antibiotic resistance genes of engineered live bacteria may be accidentally transferred to the microbiota within the host or other microorganisms in the environment. For example, *E. coli* LM715-1 carrying the broad-host-range RP4 conjugative plasmid can transfer antibiotic resistance genes through direct bacterial contact [207]. In the complex intestinal ecosystem devoid of antibiotics, this bacterium is still capable of transmitting antibiotic resistance traits to multiple native microbial communities. This may escalate the therapeutic challenges associated with bacteria that are otherwise susceptible to conventional antibiotics. To address the issue of horizontal gene transfer, researchers have proposed several measures. For instance, integrating exogenous genes into the bacterial chromosome instead of using free plasmids can enhance the genetic stability of engineered bacteria [208]. Or, strictly screening and eliminating mobile genetic elements in engineered bacteria can reduce the risk of horizontal gene transfer. Additionally, a diffusion control system can be adopted to ensure that even if the bacteria lyse, the exogenous DNA will rapidly degrade and cannot be captured by other bacteria [209], 210]. Finally, the risk of horizontal gene transfer of engineered live bacteria can also be reduced by using gene containment technology. The main methods include knocking out essential genes, making engineered bacteria dependent on exogenous nutrients, or setting up suicide switches. For example, some researchers constructed a thymine auxotrophic *Lactococcus lactis* strain by knocking out the *thyA* gene [211]. The survival ability of this strain is significantly reduced in the absence of exogenous thymine, thereby preventing environmental genes from invading the engineered bacteria.

In addition to the above considerations, the clinical translation of engineered live bacteria also needs to take into account the risk management of special genes and specific populations. Especially for those with weakened immune systems and liver cirrhosis. Patients with liver cirrhosis often have intestinal barrier dysfunction [212], intestinal bacterial overgrowth, and immune dysfunction. The dysfunction of the gut-liver axis significantly increases the risk of engineered live bacteria translocation [213]. For patients with weakened immune systems, engineered bacteria may cause bacteremia; due to their biological characteristics, these bacteria can survive and reproduce in the host with a weakened immune system and even break through the intestinal barrier to cause bacteremia. To enhance the biosafety of engineered live bacteria therapy, host toxicity tests are still needed, and the long-term retention of the bacteria in the body should be observed. For example, multiple preclinical studies have confirmed that engineered *Lactococcus lactis* and other engineered strains of different genera have not caused significant pathological damage to rat or mouse models. Further clinical studies such as the SYNB1618 trial have shown that the liver and kidney functions of the host remain normal after administration [214]. These results indicate that strict engineering design is still needed to comprehensively ensure the safety of engineered live bacteria and promote their more effective clinical translation.

### Regulatory limitations and manufacturing difficulties

The primary regulatory obstacle for engineered live bacteria lies in the fact that there is no globally unified standard for their definition and classification. The US Food and Drug Administration categorizes genetically engineered live bacteria as live biotherapeutic products, which are regulated by the Center for Biologics Evaluation and Research as recombinant biological products [215]. However, live biotherapeutic products explicitly exclude “vaccines, filterable viruses, oncolytic viruses, and organisms used as vectors to transfer genes into the host”, which brings uncertainty to the classification of the pipeline [216]. Currently, there are no relevant guidelines providing specific advice based on the site of action or therapeutic indication of live biotherapeutic products, nor are there guidelines specifically outlining the toxicological requirements for live biotherapeutic products. The strict requirements of the chemistry, manufacturing, and controls process are fundamentally at odds with the characteristics of live bacteria. For instance, a complete traceability system must be established for the strain seed bank, and it is necessary to ensure genetic consistency during the passage cycle and conduct comprehensive screening for exogenous factors [217]. However, spontaneous mutations may occur during the passage of live bacteria. Some engineered live bacteria indirectly treat diseases by interacting with the host’s microbiome ecosystem [218]. The adverse reactions they cause may highly overlap with the daily symptoms of underlying diseases such as inflammatory bowel disease, leading to regulatory difficulties. All these factors collectively impose restrictions on the regulation of engineered live bacteria.

Production and manufacturing challenges are also a difficulty point in the clinical transformation of engineered live bacteria. During the production stage, the core regulatory focus lies in dose control and production processes. Regarding dose management, inadequate regulation may lead to dose deviations: if the number of live bacteria is below the effective threshold, it may result in treatment failure or induce host immune tolerance [219]; if it exceeds the effective threshold, it may trigger cytokine storms and excessive immune activation, and even increase the risk of horizontal gene transfer [220]. Live bacteria fermentation is highly sensitive to environmental fluctuations, and the widespread problem of phage contamination throughout the industry also adds difficulties to the production of engineered live bacteria [221]. Inadequate regulation during the production process may lead to contamination or degradation of the seed bank, fermentation failure, and quality control defects. In addition, the cultivation of engineered live bacteria is also a challenge in production. For example, in the case of *Enterococcus* amplification culture, if the inoculation density is too low, the production strain may undergo genetic mutations and completely lose the ability to produce the target product [217]. Therefore, the production process must strictly follow cGMP standards, lock in key process parameters, and ensure high consistency between batches. In summary, given the characteristics of engineered live bacteria, it is urgent to formulate differentiated regulatory standards and complete production processes. Such regulatory standards and production processes can also serve as a guarantee mechanism for biosafety.

### Combined therapies of engineered bacteria

In the treatment of liver diseases, the application of engineered bacteria is shifting from a single intervention to a multi-dimensional combined strategy. By integrating engineered bacteria with prebiotics, postbiotics, or conventional drugs in a rational manner, the therapeutic effect can be synergistically enhanced and the multi-factor pathogenesis of liver diseases can be more comprehensively regulated. Firstly, prebiotics are mixtures composed of live bacteria and substrates that can be selectively utilized by host microorganisms. Their core mechanism functions through microbial community remodeling, metabolic optimization, and immune regulation [222]. In the context of liver diseases, a prebiotic combination consisting of Lactobacillus plantarum and aged garlic extract has been proven to upregulate the expression of hepatic peroxisome proliferator-activated receptor alpha and downregulate the expression of sterol regulatory element-binding protein 1c in a NAFLD rat model. This dual mechanism reduces hepatic fat accumulation and improves liver steatosis [223]. Through microbial community remodeling and metabolic optimization, synbiotics can improve multiple liver parameters. Although there is a large amount of evidence supporting the effect of synbiotics in reducing liver enzyme levels, their impact on liver fibrosis and liver steatosis has not been consistently observed or reported. Further research on composite prebiotics for liver diseases is necessary to obtain conclusive evidence.

Secondly, postbiotics are preparations comprising inanimate microorganisms and their components, soluble products, or metabolic byproducts that confer health benefits to the host, yet they lack colonization potential and sustained microbiota-modulating capacity [224]. Probiotics can colonize the intestinal microbiota and improve its structural composition; however, their efficacy is influenced by colonization rates. Probiotics primarily colonize the intestinal mucosa, but this process is also influenced by individual variability. The population is divided into resisters and permissive individuals, which mainly depends on their native gut microbiota. In terms of treatment, the therapeutic effect is most significant when probiotics achieve localized enrichment at the site of inflammation. The combined use of probiotics and postbiotics can generate synergistic effects and target multiple pathways, thereby enhancing therapeutic outcomes. Studies have shown that, in combined applications for disease treatment, this approach can reduce serum alanine aminotransferase and aspartate aminotransferase activities, improving liver function injury. Ye et al. demonstrated that postbiotics derived from *Lactobacillus plantarum* significantly reduced serum transaminase and triglyceride levels in mice with acute alcohol-induced liver injury by protecting hepatocytes from oxidative damage, improving lipid metabolism, and modulating the intestinal microbiota [225]. Furthermore, when comparing the combination of probiotics and postbiotics to single interventions, the combined approach resulted in a microbiota composition most similar to that of the normal control group, with advantages in Operational Taxonomic Unit abundance and diversity.

Finally, in the clinical treatment of liver diseases, probiotics are usually used in combination with conventional medications. This strategy enhances the therapeutic effect through pharmacokinetic regulation and pharmacodynamic synergy. A study has shown that UDCA, as an FXR inhibitor, can directly inhibit the gene expression and replication of HBV and reduce the accumulation of toxic bile acids (such as taurocholic acid). *Bifidobacterium* can convert deoxycholic acid, a bile acid, into UDCA through hydroxy sterol dehydrogenase, replenishing the body’s own UDCA. This dual regulation strengthens the balance of bile acid metabolism, reshapes the intestinal microbiota, and regulates the immune response. While *Bifidobacterium* alone insufficiently suppresses HBV DNA, and UDCA alone struggles to reduce hepatitis B surface antigen levels, their combination enhances antiviral efficacy, offering a potential pathway towards functional cure for chronic hepatitis B [226]. Synbiotics, as well as the combination of probiotics with postbiotics or conventional drugs, all demonstrate therapeutic potential for liver diseases. Despite increasing mechanistic research, clinical evidence remains insufficient. For instance, randomized controlled trials investigating postbiotics alone or in combination, as well as the long-term efficacy of probiotic-drug synergistic regimens in real-world settings, necessitate further in-depth investigation.

### Future perspectives

Therefore, it is imperative to continuously explore innovative solutions, flexibly optimize drug formulation strategies, and leverage genetic engineering approaches to achieve breakthroughs in packaging materials, delivery functionality, and site-specific targeting [227]. In addition, multi-omics analysis can be applied to the development of novel engineered live bacteria for different subtypes of liver diseases and diseases related to the gut-liver axis [228]. By combining genetic manipulation tools, gene editing technologies, and multi-omics analysis, we can continuously develop more precisely targeted live bacterial strains, optimize the particle size of oral formulations using diverse packaging materials, and enhance the therapeutic efficacy of engineered live bacteria through synergistic drug combinations [229]. On this basis, the continuous optimization and improvement of delivery systems and regulatory strategies for engineered live bacteria are expected to advance the clinical translation and therapeutic application of engineered live bacteria [230], especially in the management of liver diseases and disease related to the gut-liver axis.

## Conclusion

This review summarizes the natural live strains with therapeutic value, such as *Bifidobacterium*, EcN, and *B. subtilis*. These strains can intervene in liver diseases and diseases related to the gut-liver axis by regulating the intestinal flora and reshaping the intestinal microecology. On this basis, this article systematically summarizes the core engineering strategies and liver delivery systems of these therapeutic live bacteria, compares the performance characteristics and application boundaries of different technical routes, and provides a systematic theoretical reference for the modular design and in vivo delivery of engineered live bacteria. At the same time, this article systematically explains the application progress of engineered live bacteria in HCC, cirrhosis, NAFLD, hepatitis, and diseases related to the gut-liver axis. Although a large number of preclinical studies have fully confirmed the effectiveness and safety of engineered live bacteria, their clinical transformation process is still lagging behind, facing problems such as insufficient systematic clinical validation and the lack of disease-specific application standards. Therefore, in order to achieve the leap from basic research to clinical application of engineered live bacteria, solutions to these problems need to be sought.

## Data Availability

No datasets were generated or analysed during the current study.
